# Overexpression of endophilin A1 exacerbates synaptic alterations in a mouse model of Alzheimer’s disease

**DOI:** 10.1038/s41467-018-04389-0

**Published:** 2018-07-30

**Authors:** Qing Yu, Yongfu Wang, Fang Du, Shijun Yan, Gang Hu, Nicola Origlia, Grazia Rutigliano, Qinru Sun, Haiyang Yu, James Ainge, Shi Fang Yan, Frank Gunn-Moore, Shirley ShiDu Yan

**Affiliations:** 1 Department of Pharmacology and Toxicology and Higuchi Bioscience Center, University of Kansas, Lawrence, KS 66047 USA; 2 State Key Laboratory of Oral Diseases, National Clinical Research Center for Oral Diseases, West China Hospital of Stomatology, Sichuan University, 610041 Cheng Du, China; 3 CNR, Institute of Neuroscience, 56124 Pisa, Italy; 4 School of Psychology and Neuroscience, University of St Andrews, St Mary’s Quad Street, St Andrews, KY16 9JP UK; 5 School of Biology, Medical and Biological Sciences Building, University of St Andrews, North Haugh Street, St Andrews, KY16 9TF UK

## Abstract

Endophilin A1 (EP) is a protein enriched in synaptic terminals that has been linked to Alzheimer’s disease (AD). Previous in vitro studies have shown that EP can bind to a variety of proteins, which elicit changes in synaptic transmission of neurotransmitters and spine formation. Additionally, we previously showed that EP protein levels are elevated in AD patients and AD transgenic animal models. Here, we establish the in vivo consequences of upregulation of EP expression in amyloid-β peptide (Aβ)-rich environments, leading to changes in both long-term potentiation and learning and memory of transgenic animals. Specifically, increasing EP augmented cerebral Aβ accumulation. EP-mediated signal transduction via reactive oxygen species (ROS)/p38 mitogen-activated protein (MAP) kinase contributes to Aβ-induced mitochondrial dysfunction, synaptic injury, and cognitive decline, which could be rescued by blocking either ROS or p38 MAP kinase activity.

## Abstract (web summary)

Endophilin A1 protein is known to be elevated in Alzheimer’s disease (AD). Here the authors show that endophilin A1 overexpression exacerbates synaptic deficits in a mouse model of AD.

## Introduction

Progressive neuronal transmission deregulation, synaptic and neuronal loss, and declined cognition are features of Alzheimer’s disease (AD)^1–8^. Amyloid-β peptide (Aβ) is one of the critical molecular factors in AD pathogenesis and causes synapse deterioration in the early stages of AD^9–12^. Specifically, Aβ deregulates neurotransmitter release from the presynaptic site from studies both in vitro with oligomer Aβ-treated primary neuronal cultures and in vivo AD mouse models overexpressing amyloid precursor protein (APP)/Aβ^13–15^. Subsequently, the post-synaptic dependent long-term synaptic plasticity is affected by Aβ. These changes in synaptic transmission events are associated with the loss of synapses, neuronal perturbations, and memory decline in AD. However, the molecular mechanisms for these deleterious effects of Aβ on synaptic transmission events and specifically those relevant to the critical neurotransmitter release/recycling machinery, have not been reported.

Endophilin A1 (EP) is a brain-specific protein enriched in synaptic terminals^16^. It has been reported to bind with synaptojanin, synaptotagmin, synaptosomal-associated protein 25, and vesicle glutamate transporter 1, which in turn are directly involved in neurotransmitter release. EP also plays a key role in endocytosis, which is a critical process for the clearance of neurotransmitters from synaptic cleft and dendritic spine morphogenesis and stability^17–19^. The interaction of EP with synaptojanin is required for synaptic vesicle endocytosis by retrieval of synaptic vesicles^20^. Therefore, EP is a crucial molecular player in terms of governing synaptic transmission. Other studies indicate that loss of EP function in mice leads to neuronal dysfunction under normal physiological condition^21,22^, and its expression can control glutamate release^23^ and affects dendritic spine formation^19^.

Although the important role of EP in synaptic transmission was first established in the past decade, only a few studies have illustrated EP as a mediator for synaptic malfunction in neurodegenerative diseases. Intriguingly, a role of EP in synaptic dysfunction and neuronal loss in Parkinson disease has been reported^17,24–27^. For example, in the Parkinson disease-affected brain, EP interacts with leucine-rich repeat kinase 2 (LRRK2) and parkin, serving as a substrate that can be modified by phosphorylation or ubiquitination, which results in synaptic dysfunction and loss^22,25^. With respect to AD, we have previously demonstrated that EP is significantly increased in AD-affected brain regions when compared to the non-AD brain. In addition, we showed that EP levels were also higher in Aβ-rich brains from transgenic (Tg) AD mice again when compared to non-Tg control mice^28^, thus suggesting that EP may potentially be an important intracellular player in the synaptic alterations detected in AD pathogenesis. However, to date, the direct effects of EP on Aβ-induced synaptic impairment in vivo AD mice have not yet been explored.

In the present study, we generated and characterized Tg mice overexpressing EP in neurons. Using this genetically manipulated neuronal EP mouse model and a neuronal culture system with an Aβ-enriched environment, we have comprehensively analyzed the effects of neuronal EP on Aβ-induced abnormalities in synaptic neurotransmission and plasticity, synaptic density, and also the altered learning and memory capabilities. We were also interested in synaptic mitochondria as they are vital for providing energy and modulating calcium homeostasis as well as being the main resource for the generation of reactive oxygen species (ROS). Consequently, we analyzed the effect of EP on mitochondrial function and oxidative stress to determine whether EP-mediated mitochondrial defect links to synaptic alterations caused by Aβ insult. As we had previously shown that EP could affect the stress kinases^28^, we also assessed how EP could affect the oxidative stress and relevant signaling pathway via activation of p38 mitogen-activated protein (MAP) kinase. In view of the impact of ROS on Aβ metabolism, we finally analyzed the effect of EP on cerebral Aβ accumulation and APP processing. Our studies indicate that EP signaling does contribute to amyloid pathology and Aβ-induced synaptic injury and impairment in learning and memory in AD.

## Results

### Tg mice overexpressing neuronal EP

In view of that EP has a raised expression in the brains of AD patients^28^ and Tg mice with neuronal overexpression of a mutant human form of APP (Tg mAPP, APPSwInd, J-20 line) driven by the platelet-derived growth factor β-chain promoter at 9–10 months of age (Supplementary Fig. 1a, b), we sought to develop a model system in which neuronal expression of EP would be exaggerated so that consequences of EP-dependent signaling in Aβ-rich environment could be established. A transgene bearing full-length mouse EP driven by Thy-1 promoter was constructed and used to generate Tg mice, termed Tg *Sh3gl2* (Supplementary Fig. 2a). Tg *Sh3gl2* mice were identified as bearing the transgene by polymerase chain reaction (PCR) analysis of tail DNA (Supplementary Fig. 2b). Immunoblotting of cortical homogenates confirmed the increase in EP expression in Tg *Sh3gl2* mice, compared with non-Tg littermates (Supplementary Fig. 2c, d). Immunostaining of brain sections demonstrated enhanced expression of EP antigen in cortical and hippocampal neurons of Tg *Sh3gl2* mice compared with non-Tg littermates (Supplementary Fig. 2e–g).

### EP expression aggravates Aβ-induced LTP reduction

**Fig. 1:**
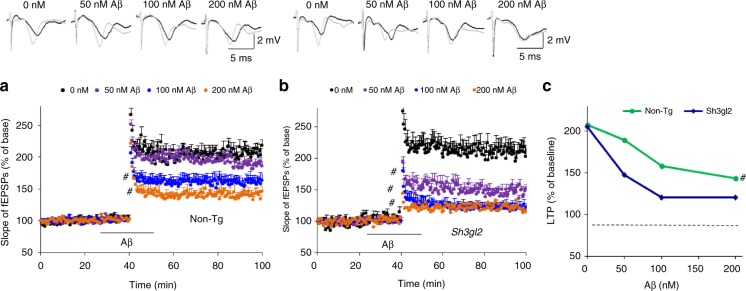
Effect of EP overexpression on oligomer Aβ-induced hippocampal synaptic deficit. Hippocampal slices were derived from 3-month-old non-Tg and Tg *Sh3gl2* mice. Slices were perfused with 50, 100, and 200 nM Aβ for 1 h**. a** Fifty nanomolar of oligomer Aβ perfusion over non-Tg slices does not significantly reduce LTP level. Perfusion of non-Tg hippocampal slices with higher Aβ concentration (100 nM, 200 nM) impairs hippocampal LTP expression. Error bars represent s.e.m., *n* = 7–10 per group; ^#^*p* < 0.05 (one-way ANOVA). **b** Fifty nanomolar of oligomer Aβ perfusion over Tg *Sh3gl2* slices significantly reduce the LTP level. Perfusion with higher Aβ concentration (100 nM, 200 nM) saturated the deleterious effect of Aβ on hippocampal LTP impairment. Line indicates Aβ exposure period. Error bars represent s.e.m., *n* = 7–10 per group; ^#^*p* < 0.05 (one-way ANOVA). **c** Upper panels of a and b show representative traces of fEPSP in slices with the indicated treatment before θ-burst stimulation (black line) and after 1 h (gray line). Summarized LTP levels (average of the fEPSPs slope of the last 10 min recordings) in the indicated groups. Data are shown as mean ± s.e.m., *n* = 7–10 per group; ^#^*p* < 0.05 (Student’s *t* test)

We first determined whether increased EP expression aggravated Aβ-induced synaptic dysfunction by recording long-term potentiation (LTP) in hippocampal CA1 neurons from EP overexpression mice (Tg *Sh3gl2*) and non-Tg littermate controls. Hippocampal slices from 3-month-old non-Tg and Tg *Sh3gl2* mice were exposed to a variety of oligomer Aβ concentrations (50, 100, and 200 nM): non-Tg slices displayed a significant decrease in LTP from baseline to 188%, 158%, and 148%, respectively, whereas Tg *Sh3gl2* slices demonstrated a further reduction in LTP 147%, 120%, and 120%, respectively (Fig. 1a–c). The basal synaptic transmission (BST) was unchanged either in non-Tg or Tg *Sh3gl2* hippocampal slices (Supplementary Fig. 3a, b). These results indicate that increased neuronal EP exacerbates synaptic impairment induced by Aβ.

### EP impairs synaptic function, learning, and memory

Again, given that EP expression was significantly elevated in human AD brains enriched for Aβ accumulation, Tg *Sh3gl2* mice were crossed with Tg mAPP mouse^29^ to mimic an AD environment. Tg mAPP is a well-known AD mouse model, and has been well characterized with respect to neuropathology, synaptic, and cognitive function^11,13^. Thus, this AD mouse model was well suited for our strategy of determining whether overexpression of EP might enhance/accelerate Aβ-induced synaptic dysfunction and learning and memory impairments in an in vivo setting. Tg *Sh3gl2* mice were cross-bred with Tg mAPP mice to produce double Tg mice (*Sh3gl2*/mAPP), single Tg mice (*Sh3gl2*, mAPP), and non-Tg littermate controls.

**Fig. 2:**
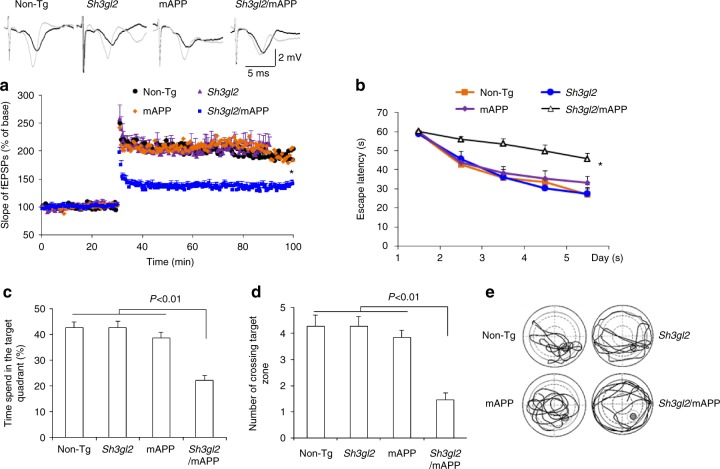
Effect of EP overexpression on synaptic plasticity and spatial learning and memory in transgenic Tg *Sh3gl2*/ mAPP mice. **a** Tg *Sh3gl2* or mAPP at 5–6 months of age do not alter hippocampal LTP, but the hippocampal LTP is significantly reduced in Tg *Sh3gl2*/mAPP mice as compared with non-Tg mice. Error bars represent s.e.m., *n* = 7–10 per group; *p < 0.01 (one-way ANOVA). Upper panel shows representative traces of fEPSP in the indicated slices before θ-burst stimulation (black line) and after 1 h (gray line). **b**–**e** Mice were tested in Morris water maze at the age of 5–5.5 months. **b** Escape latencies in hidden platform during Morris water maze task training in indicated groups of mice. Error bars represent s.e.m., *n* = 8–9 mice per group (one-way ANOVA in **b**). **c** Time spent in the quadrant with the hidden platform and **d** mean number of crossings of the target during the probe test. **e** The representative searching traces during the probe test. Data are shown as mean ± s.e.m., *n* = 8–9 mice per group (one-way ANOVA in **c**, **d**)

Utilizing these new Tg animals, we first examined synaptic transmission under basal conditions and during LTP. Compared to other groups of mice, Tg Sh3gl2/mAPP mice revealed significant reduction in CA1 neuronal LTP (Fig. 2a). There were no changes in BST as shown by field-excitatory post-synaptic potential (fEPSPs) and LTP between single Tg mice (Tg *Sh3gl2* and mAPP mice) and non-Tg control mice at 5–6 months of age (Supplementary Fig. 3c).

We next evaluated whether these EP-induced deficits in synaptic activity were also reflected in behavioral changes. Mice were subjected to Morris water maze (MWM) for evaluation of the spatial learning and memory. Although behavioral testing results obtained in different laboratories can vary^30,31^ due to the testing protocol and variable environment, our results from hidden platform MWM test are consistent with results from ours and others previously published reports, which showed that deficits in learning and memory in Tg mAPP mice occurred at 6–7 months of age or later^13,32,33^ compared to non-Tg mice. Notably, Tg *Sh3gl2*/mAPP mice displayed a significantly longer latency to locate the hidden platform during the training session (Fig. 2b) and decreased time spent in the target area (Fig. 2c) and the number of times crossing the target (Fig. 2d, e) during the recording period in comparison with mAPP mice. Thus, Tg *Sh3gl2*/mAPP mice exhibited exacerbated impairments in spatial learning and memory compared to mAPP mice. The different groups of Tg mice had similar swimming speeds as established by the visual swimming speed test (Supplementary Fig. 4a). Thus, the observed difference in spatial learning and memory of Tg *Sh3gl2*/mAPP mice is a result of cognitive decline, which is not due to alteration in motility or motivation. These data indicate that increased neuronal EP expression accelerates and exaggerates synaptic abnormality and learning and memory impairments in mAPP mice.

### EP aggravates ROS and mitochondrial dysfunction

**Fig. 3:**
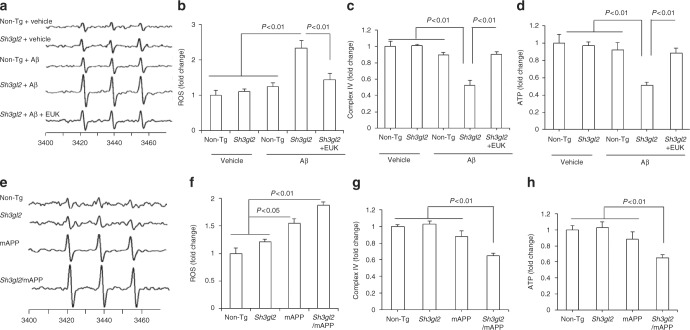
Effect of antioxidant on EP/Aβ-mediated ROS production and mitochondrial dysfunction in brain in vivo and brain slices in vitro. **a** The peak height in the spectrum indicates the levels of ROS. Representative spectrum of EPR in indicated mice brain slices perfused with Aβ or vehicle in the presence/absence of antioxidant EUK-134 (EUK). Brain slices from indicated Tg mice were pretreated with EUK (500 nM) for 5 min before Aβ perfusion (50 nM for 1 h). **b** Data are presented as fold increase relative to vehicle-treated non-Tg mice slices. Mitochondrial complex IV activity (**c**) and ATP levels (**d**) were demonstrated in the indicated hippocampus treated with vehicle or Aβ in the presence/absence of EUK. Date are shown as mean ± s.e.m., *n* = 3 per group (one-way ANOVA in **b**–**d**). **e** Representative spectra of EPR in the indicated Tg mice at 5–6 months of age . **f** Quantification of EPR spectra in the indicated mice brain. Data are expressed as fold increase relative to non-Tg mice. Date are shown as mean ± s.e.m., *n* = 5 mice per group (one-way ANOVA in **f**). Mitochondrial complex IV activity (**g**) and ATP levels (**h**) in indicated mice were assayed. Data are shown as mean ± s.e.m., *n* = 6–10 mice per group (one-way ANOVA in **g–h**)

Because Aβ facilitates oxidative stress-induced neuronal dysfunction and synaptic injury^13^ and because oxidative stress and Aβ alter EP expression levels^28^, we determined whether increased EP expression enhanced Aβ-mediated generation of reactive oxygen free radicals (ROS). Using highly specific and sensitive electron paramagnetic resonance (EPR) spectroscopy, we quantitatively measured ROS levels in brain slices from 3-month-old mice in response to Aβ. Fifty nanomolar of Aβ-treated non-Tg slices did not show an increase in ROS levels compared to vehicle-treated slices. However, exposure of Tg *Sh3gl2* slices to 50 nM Aβ produced higher levels of EPR spectra than Aβ-treated non-Tg slices (Fig. 3a, b). Application of antioxidant EUK-134 (EUK, 500 nM), a synthetic superoxide dismutase/catalase mimetic, diminished Aβ-induced ROS accumulation (Fig. 3a, b). Only Tg *Sh3gl2* brain slices with 50 nM Aβ treatment showed significant mitochondrial dysfunction as demonstrated by the reduction in mitochondrial respiratory chain key enzyme CcO (cytochrome c oxidase) activity and ATP levels. The addition of antioxidant EUK blocked EP-mediated mitochondrial defect (Fig. 3c, d). Consistent with these in vitro results with Aβ treatment, double Tg *Sh3gl2*/mAPP mice displayed a significant higher oxidative stress and worse mitochondrial function than the other groups of mice, including Tg mAPP, Tg *Sh3gl2*, and non-Tg littermates (Fig. 3e–h). These results indicate that overexpression of neuronal EP enhances Aβ-induced ROS generation, accumulation, and mitochondrial dysfunction. To confirm the effect of neuronal EP overexpression on Aβ-induced oxidative stress and mitochondrial dysfunction, we evaluated mitochondrial function by assessing ROS levels, CcO activity, and ATP levels in EP-overexpressed or non-Tg neurons cultured from Tg *Sh3gl2* mice or non-Tg mice, respectively. Tg *Sh3gl2* neurons with Aβ treatment revealed a significant elevated ROS level and declines in CcO activity and ATP levels (Supplementary Fig. 5a–d); in contrast, non-Tg-derived neurons with the same treatment did not show such changes (Supplementary Fig. 5a–d).

### EP activates p38 MAP kinase signaling

**Fig. 4:**
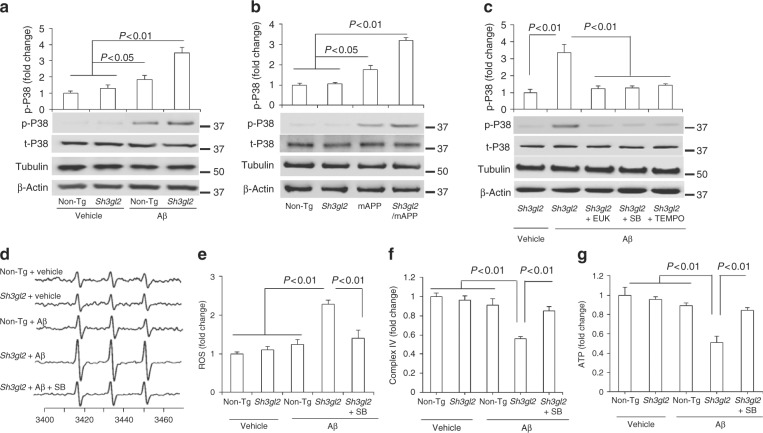
Effect of EP overexpression on p38 MAP kinase activation and mitochondrial dysfunction in Aβ-insulted brain in vivo and brain slices in vitro. **a** Brain slices from 3-month-old non-Tg or Tg *Sh3gl2* mice were perfused with Aβ (50 nM) or vehicle for 1 h and then subjected to immunoblotting analysis for the phosphorylation of p38 MAP kinase (p-p38), total p38 MAP kinase (t-p38), tubulin, and β-actin. Tubulin and β-actin served as a neuronal marker and protein loading controls, respectively. Data are expressed as fold change relative to the non-Tg vehicle control group. **b** Immunoblotting of cortical homogenates from the indicated Tg mice at 5–6 months of age for the indicated proteins. Data are expressed as fold change relative to the non-Tg mice group. **c** Brain slices from indicated Tg *Sh3gl2* mice were treated with vehicle or Aβ (50 nM) with/without pretreatment of EUK-134 (EUK, 500 nM), SB203580 (SB, 1 µM), or mitochondrial antioxidant MitoTEMPO (TEMPO, 1 µM) for 5 min, and then subjected to immunoblotting for the phosphorylation of p38 MAP kinase (p-P38), total p38 MAP kinase (t-p38), tubulin, and β-actin. Data are expressed as fold change relative to the Tg *Sh3gl2* vehicle control group. Date are shown as mean ± s.e.m., *n* = 3 per group (one-way ANOVA in **a**–**c**). **d** Representative spectra of EPR in non-Tg and Tg *Sh3gl2* brain slices with the treatment of vehicle or Aβ (50 nM) in the presence of SB203580 (1 µM). **e** Quantification of EPR spectra in the indicated groups of mice. **f**–**g** Mitochondrial complex IV activity (**f**) and ATP levels (**g**) in the indicated groups of brain slices treated with vehicle or Aβ in the presence/absence of SB203580. Data are expressed as fold increase relative to non-Tg vehicle control group. Date are shown as mean ± s.e.m., *n* = 3 per group (one-way ANOVA in **e**–**g**)

Aβ and oxidative stress both induce activation of p38 MAP kinase, and its phosphorylation has been demonstrated to link neuronal and synaptic perturbation^34–36^. Therefore, we next evaluated the potential role of p38 MAP kinase activation in EP-involved synaptic damage, using antibodies to phosphorylate p38 MAP kinase and hippocampal extracts to estimate activation. Immunoblotting of hippocampal lysates exhibited that phosphorylation of p38 MAP kinase occurred selectively in Aβ-perfused slices as compared to vehicle-treated slices, whereas the level of phosphorylation of p38 MAP kinase was significantly higher in Tg *Sh3gl2*-derived slices than non-Tg slices in the presence of Aβ (Fig. 4a). Levels of total p38 MAP kinase were comparable between non-Tg and Tg *Sh3gl2* slices with or without treatment of Aβ. Consistent with in vitro results with Aβ treatment, double Tg *Sh3gl2*/mAPP mice also displayed a significant higher level of phosphorylation of p38 MAP kinase than the other groups of mice, including Tg mAPP, Tg *Sh3gl2*, and non-Tg littermates (Fig. 4b). These results indicate that EP is involved in Aβ-induced activation of p38 MAP kinase signal transduction.

**Fig. 5:**
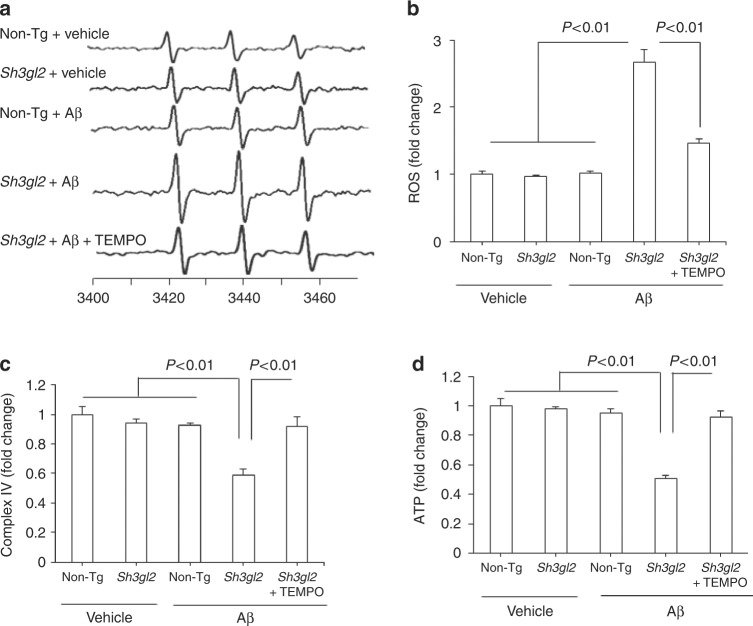
Effect of mitochondrial ROS scavenger on EP/Aβ-mediated p38 activation, ROS production, and mitochondrial dysfunction. **a** Brain slices from 3-month-old non-Tg or Tg *Sh3gl2* mice were perfused with Aβ (50 nM) for 1 h with/without pretreatment of mitochondrial antioxidant MitoTEMPO (TEMPO, 1 µM) for 5 min, and then subjected to measure EPR, mitochondrial complex IV activity, and ATP levels. **a** Representative spectrum of EPR in non-Tg and Tg *Sh3gl2* brain slices with the treatment of vehicle or Aβ in the presence of MitoTEMPO. **b** Quantification of EPR spectra in the indicated groups of mice. **c**, **d** Mitochondrial complex IV activity (**c**) and ATP levels (**d**) in the indicated groups of brain slices treated with vehicle or Aβ in the presence/absence of MitoTEMPO. Data are expressed as fold change relative to non-Tg vehicle control group. Date are shown as mean ± s.e.m., *n* = 3 per group (one-way ANOVA in **b**–**d**)

To determine whether EP/Aβ-mediated ROS provokes the activation of p38 MAP kinase signal transduction, Tg *Sh3gl2* slices were first treated with antioxidant EUK, MitoTEMPO, or p38 MAP kinase inhibitor (SB203580) for 5 min prior to the addition of Aβ. The addition of SB or antioxidant EUK inhibited phosphorylation of p38 MAP kinase (Fig. 4c), along with suppressed ROS, and completely restored CcO activity and ATP levels (Fig. 4d–g) in the presence of Aβ. These results suggest that EP-mediated p38 MAP kinase activation is responsible for Aβ-induced aberrant mitochondrial function and oxidative stress. Furthermore, by applying a mitochondria-targeted antioxidant MitoTEMPO (TEMPO) in Aβ-treated Tg *Sh3gl2* slices, phosphorylation of p38 MAP kinase was inhibited (Fig. 4c) along with the suppression of ROS levels (Fig. 5a, b), increased CcO activity and ATP levels (Fig. 5c, d), implying that elevated mitochondrial oxidative stress induced by EP/Aβ contributes to mitochondrial alterations. Moreover, addition of the antioxidant EUK or the specific p38 MAP kinase inhibitor, SB203580/SB, not only markedly reduced ROS levels but also rescued mitochondrial dysfunction in Aβ treated Tg *Sh3gl2* neurons (Supplementary Fig. 5e–h). These results confirmed that increased EP expression enhances Aβ-induced oxidative stress and mitochondrial dysfunction, which can be rescued by antioxidant and p38 MAP kinase inhibitors.

**Fig. 6:**
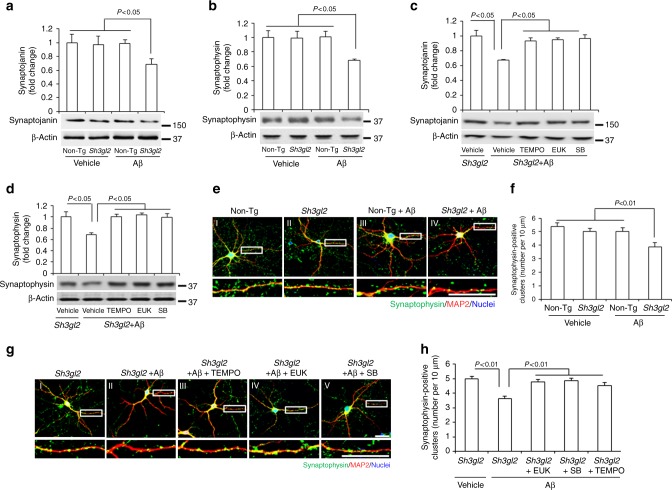
Blocking EP-mediated oxidative stress and p38 activation rescued Aβ-induced synaptic loss. **a**, **b** Brain slices from 3-month-old non-Tg or Tg *Sh3gl2* mice were perfused with Aβ (50 nM) for 2 h, and then subjected to immunoblotting analysis for synaptojanin (**a**) and synaptophysin (**b**) in the indicated groups of brain slices. β-Actin served as protein loading controls. The upper panel displays quantification of immunoreactive bands for the corresponding protein relative to β-actin. Data are expressed as fold change relative to the non-Tg vehicle control group. Data are shown as mean ± s.e.m., *n* = 3 per group (one-way ANOVA in **a**, **b**). **c**, **d** The Tg *Sh3gl2* brain slices from 3-month-old mice were perfused with Aβ (50 nM) for 2 h with/without pretreatment of 500 nM EUK-134 (EUK), 1 μM SB203580 (SB), or 1 µM MitoTEMPO (TEMPO) for 5 min. Immunoblotting for synaptojanin (**c**) and synaptophysin (**d**) in the indicated groups of brain slices. The upper panel displays the quantification of immunoreactive bands for the corresponding protein relative to β-actin. Data are expressed as fold change relative to Tg *Sh3gl2* vehicle control group. Data are shown as mean ± s.e.m., *n* = 3 per group (one-way ANOVA in **c**, **d**). Fourteen-day in vitro cultured cortical neurons, either non-Tg or Tg *Sh3gl2*, were treated with 50 nM Aβ for 24 h, with or without 500 nM EUK-134, 1 μM SB203580, or 1 μM MitoTEMPO pretreatment for 1 h before the addition of Aβ. The numbers of synaptophysin-positive clusters were significantly decreased in Aβ-treated Tg *Sh3gl2* neurons compared to vehicle-treated non-Tg neurons in **e**–**h**. Treatment with EUK-134, or SB203580, or MitoTEMPO, inhibited Aβ-induced synaptic loss in cultured EP overexpression neurons (**g**, **h**). Representative images for synaptophysin (green), MAP2 (red), and nuclei (blue) in the indicated groups of neurons are shown in **e**, **g**. Scale bars, 50 μm. Quantifications of synaptophysin-positive clusters per 10 μm of dendrites are shown in **f**, **h**. Data are shown as mean ± s.e.m., *n* = 12 cells for each group (one-way ANOVA in **f**, **h**)

Recent studies have shown a link between EP expression and spine morphogenesis. Therefore, we next assessed the direct effect of neuronal EP on Aβ-induced synaptic protein loss and morphology by quantification of synaptic protein levels and synaptic density. Both presynaptic proteins synaptojanin and synaptophysin were significantly reduced in Tg *Sh3gl2* hippocampal slice exposed to Aβ (50 nM) compared to Aβ-treated non-Tg or vehicle-treated Tg *Sh3gl2* slices (Fig. 6a, b). Treatment of antioxidant EUK, TEMPO, or the p38 MAP kinase inhibitor (SB203580) prevented loss of these presynaptic proteins in EP/Aβ-insulted slices (Fig. 6c, d). Synaptic density was quantified by measuring synaptophysin-positive clusters attaching to dendrites labeled with MAP2. Non-Tg neurons treated with a low concentration of Aβ (50 nM) did not exhibit loss of synapses compared to vehicle-treated cells (Fig. 6e, f). However, Tg *Sh3gl2* neurons had a significantly decreased synaptic density (Fig. 6e–h). Importantly, scavenging ROS by the addition of EUK-134 (Fig. 6g–h) or MitoTEMPO (Fig. 6g–h), or inhibiting p38 MAP kinase activation (Fig. 6g–h) effectively protected against Aβ-induced synaptic loss. These results suggest that suppression of ROS-involved activation of p38 MAP kinase signaling rescues EP/Aβ-induced synaptic loss.

**Fig. 7:**
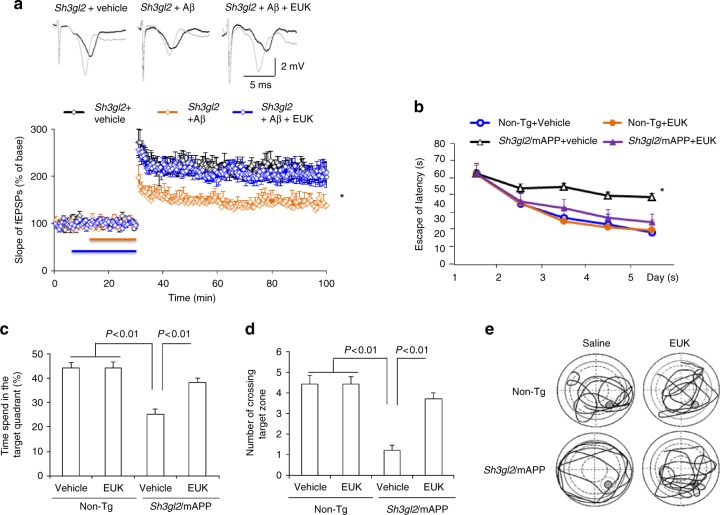
Effect of ROS scavenger on EP/Aβ-mediated synaptic plasticity and spatial learning and memory impairment. **a** Hippocampal slices from 5-month-old to 6-month-old Tg *Sh3gl2* mice were pretreated with EUK-134 (500 nM) for 5 min before Aβ perfusion (100 nM for 20 min), and then hippocampal CA3-CA1 LTP was recorded. Error bars represent s.e.m., *n* = 7–10 per group. *p < 0.01 (one-way ANOVA). Non-Tg and Tg *Sh3gl2*/mAPP mice were intraperitoneal injected with EUK-134 (2 mg/kg) once a day for 3 weeks and then performed a Morris water maze test at 5–5.5 months of age. Upper panel shows representative traces of fEPSP in slices with the indicated treatment before θ-burst stimulation (black line) and after 1 h (gray line). **b** Escape latencies in hidden platform during Morris water maze task training in indicated groups. Error bars represent s.e.m., *n* = 8–9 per group. *p < 0.01 (one-way ANOVA). **c** Time spent in the quadrant with the hidden platform and **d** mean number of crossings of the target during the probe test. **e** Representative searching traces during the probe test. Learning and memory were impaired in Tg *Sh3gl2*/mAPP mice compared to other groups, which was rescued by antioxidant EUK treatment. Data are shown as mean ± s.e.m., *n* = 8–9 mice per group (one-way ANOVA in **c**, **d**)

To further evaluate whether EP/Aβ-induced oxidative stress is responsible for the deficits in synaptic plasticity, Tg *Sh3gl2* hippocampal slices were treated with the antioxidant EUK-134 in the presence of Aβ. Treatment with EUK-134 completely restored the EP/Aβ-induced hippocampal LTP decline (Fig. 7a). The BSTs were unchanged in the indicated Tg *Sh3gl2* hippocampal slices (Supplementary Fig. 7a). Similarly, administration of EUK-134 to Tg *Sh3gl2*/mAPP mice revealed the improvement in spatial learning and memory, showing shorter latency to find the platform during training (Fig. 7b), longer time spent in the target area (Fig. 7c), and an increase in the number of times crossing the target (Fig. 7d, e) in the MWM behavioral test. The swimming speed was comparable among the indicated four groups of mice (Supplementary Fig. 4b). These results indicate that blockade of EP/Aβ-mediated oxidative stress improves synaptic and cognitive function.

### Inhibition of p38 MAP kinase rescues synaptic deficits

**Fig. 8:**
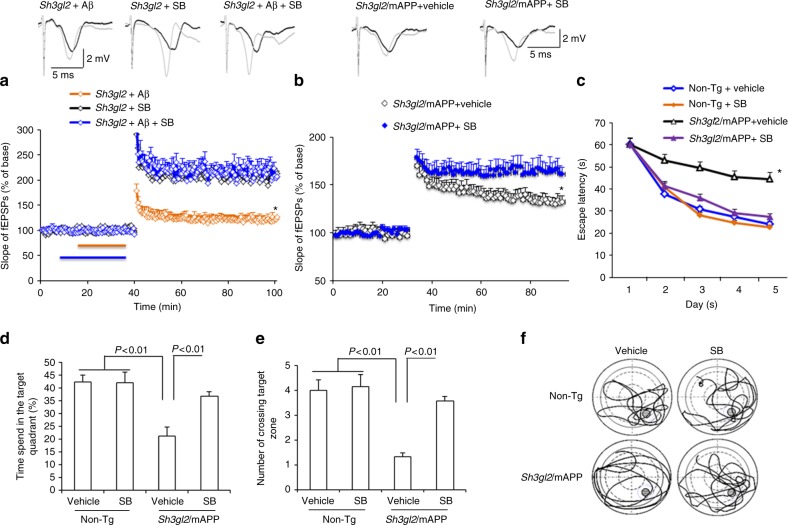
Inhibition of p38 MAP kinase rescues impairment on synaptic plasticity and spatial learning and memory in Tg *Sh3gl2*/mAPP mice. **a**, **b** Hippocampal slices from 5-month-old to 6-month-old Tg *Sh3gl2* mice were pretreated with SB203580 (SB, 1 µM) for 5 min before Aβ perfusion (100 nM for 20 min) and then hippocampal CA3-CA1 LTP was recorded (**a**). Tg *Sh3gl2*/mAPP mice were intraperitoneally injected with SB203580 (0.5 mg/kg) once a day for 3 weeks and then performed LTP experiments (**b**) and Morris water maze test (**c**–**f**) at the age of 5–5.5 months. Upper panels of a and b show representative traces of fEPSP in the indicated slices with the indicated treatment before θ-burst stimulation (black line) after 1 h (gray line). Administration of SB203850 significantly ameliorated hippocampal LTP deficit in Tg *Sh3gl2*/mAPP mice compared to the vehicle-treated group. Error bars represent s.e.m., *n* = 7–10 per group. *p < 0.01 (one-way ANOVA in **a**, **b**). **c** Escape latencies in hidden platform during Morris water maze task training in indicated groups. Error bars represent s.e.m., *n* = 8–9 mice per group (one-way ANOVA). **d** Time spent in the quadrant with the hidden platform and **e** mean number of crossings of the target during .the probe test. **f** Representative searching traces during the probe test. Data are shown as mean ± s.e.m., *n* = 8–9 mice per group (one-way ANOVA in **d**, **e**)

Next, we evaluated whether the p38 MAP kinase pathway was involved in the EP-mediated deficits in synaptic plasticity instigated by Aβ. Tg *Sh3gl2* hippocampal slices were treated with SB203580 in the presence of Aβ. Blockade of p38 MAP kinase activity completely restored hippocampal LTP in Tg *Sh3gl2* mice (Fig. 8a). The BSTs were unchanged in the indicated Tg *Sh3gl2* hippocampal slices (Supplementary Fig. 7b).

To further confirm the effect of EP-mediated p38 MAP kinase signaling in vivo Tg mAPP mice, Tg *Sh3gl2*/mAPP mice were administrated with SB203580 (0.5 mg/kg, daily) for 3 weeks and then evaluated for LTP and learning and memory. Tg *Sh3gl2*/mAPP mice, which received the p38 MAP kinase inhibitor, showed an increase in LTP as compared to vehicle-treated mice (Fig. 8b). The BSTs were unchanged in the indicated Tg *Sh3gl2*/mAPP hippocampal slices (Supplementary Fig. 7c). Similarly, treatment of SB203580 significantly improved spatial learning and memory as shown by shorter latency to find the platform during training (Fig. 8c) and increased the time spent in the target quadrant and the number of times crossing the target (Fig. 8d–f) during the recording period. The swimming speed was comparable among the indicated four groups of mice (Supplementary Fig. 4c). These results indicate that blockade of EP-mediated activation of p38 MAP kinase improves synaptic and cognitive function.

### EP impairs Aβ-induced synaptic vesicle recycling

**Fig. 9:**
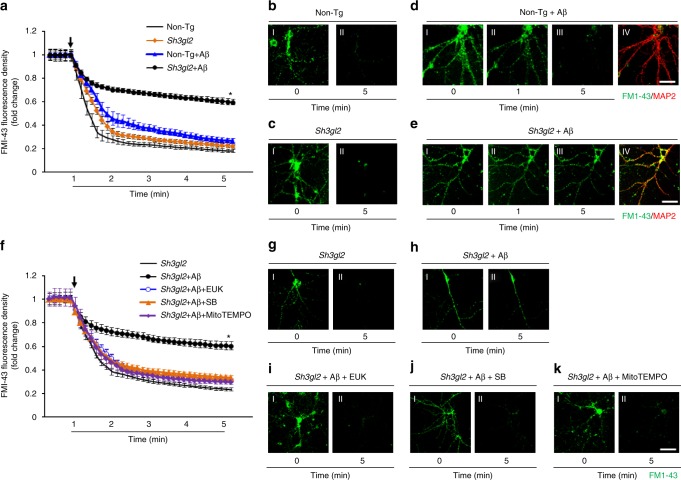
Blocking EP-mediated oxidative stress and p38 activation rescued Aβ-induced synaptic vesicle recycling impairment. Fourteen-day in vitro cultured cortical neurons, either non-Tg or Tg *Sh3gl2*, were treated with 50 nM Aβ for 24 h, with or without 500 nM EUK-134, 1 μM SB203580/SB, or 1 μM MitoTEMPO pretreatment for 1 h before the addition of Aβ. To visualize synaptic vesicle recycling, the cells were loaded with the fluorescent styryl dye FM1–43 before and after stimulation with 50 mM K^+^ for the indicated time. **a**, **f** Kinetics of FM1–43 unloading of synaptic boutons during sustained stimulation with 50 mM KCl. **b–e** Fluorescence images before (I) and after (II, III) FM1–43 unloading with 50 mM KCl, and the representative immunofluorescence images of MAP2 (red, IV) to ensure the position of FM1–43 fluorescence (green, IV in **d**, **e**). Tg *Sh3gl2* neurons treated with 50 nM Aβ for 24 h alone (**e**, **h**) showed synaptic vesicle release impairment compared to the vehicle Tg *Sh3gl2* treatment (**c**, **g**) and non-Tg neurons, whereas treatment with 50 nM Aβ (**d**) showed no difference compared to the vehicle non-Tg neurons (**b**). Pretreatment with 500 nM EUK-134 (**i**), 1 μM SB203580 (**j**), or 1 μM MitoTEMPO (**k**) rescued Aβ-induced synaptic vesicle recycling impairment in Tg *Sh3gl2* neurons. Scale bar = 50 µm. Error bars represent s.e.m., *n* = 8 per group. *p < 0.01 compared to other groups in **a** and **f** (one-way ANOVA)

To evaluate the effect of EP on Aβ-induced synaptic vesicle recycling, we investigated the capacity of synaptic vesicle release. To visualize synaptic vesicle recycling, 14-day in vitro cultured cortical neurons were loaded with the fluorescent styryl dye FM1–43 as a marker for synaptic vesicles (Fig. 9bI–eI and gI–kI). Synaptic vesicle release was indicated by the disappearance of FM1–43 fluorescent intensity upon stimulation with 50 mM K^+^ (Fig. 9bII–eII, dIII–eIII, and gII–kII). The fluorescent density was normalized by dividing the initial fluorescence prior to the addition of K^+^ in each nerve terminal, and the kinetics of fluorescent styryl dye FM1–43 loss was assessed from randomly selected synaptic boutons (Fig. 9a, f). The unloading phase contains the rapid release of dye from a mobilizable pool of vesicles, and the slow replenishment of this rapidly mobilizable vesicle population is from the reserve pool^37^. In non-Tg neurons, synaptic boutons exhibited a strong dye loss (Fig. 9a, b), whereas treatment with Aβ induced a weak dye loss from synaptic boutons (Fig. 9a, d). In contrast, a much weaker dye loss was detected in synaptic boutons from Tg *Sh3gl2* neurons with Aβ treatment (Fig. 9a, e), although there was no significant difference between non-Tg and Tg *Sh3gl2* neurons with vehicle treatment (Fig. 9a–c). This indicates that Aβ impairs synaptic vesicle recycling ability in Tg *Sh3gl2* neurons. Interestingly, administration of EUK-134 (Fig. 9f, i), SB203580 (Fig. 9f, j), or MitoTEMPO (Fig. 9f, k) completely rescued this synaptic vesicle recycling impairment in Tg Sh3gl2 neurons treated with Aβ, suggesting that EP-involved oxidative stress and the p38 MAP kinase signal pathway are responsible for cerebral synaptic vesicle recycling impairment in an Aβ-rich environment.

### EP promotes Aβ accumulation in Tg mAPP mice

**Fig. 10:**
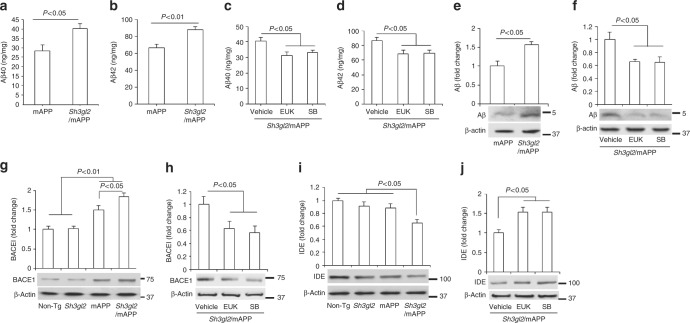
Effect of EP overexpression on cerebral Aβ accumulation. ELISA for measurement of Aβ40 (**a**, **c**) and Aβ42 (**b**, **d**) in the entorhinal cortex of Tg mAPP and Tg *Sh3gl2*/mAPP mice at the age of 5–5.5 months. EUK-134 (EUK, 2 mg/kg) (**c**, **d**) or SB203580 (SB, 0.5 mg/kg) (**c**, **d**) was administered to Tg *Sh3gl2*/mAPP mice once a day for 3 weeks and then cortical tissues were subjected to Aβ measurement at the age of 5–5.5 months. Date are shown as mean ± s.e.m., *n* = 3–6 per group (one-way ANOVA in **a–d**). Quantification of immunoreactive bands for Aβ (**e**), BACE1 (**g**), or IDE (**i**) in the indicated Tg mice at the age of 5–5.5 months. Quantification of immunoreactive bands for Aβ (**f**), BACE1 (**h**), or IDE (**j**) in Tg *Sh3gl2*.mAPP mice treated with EUK or P38 inhibitor (SB) relative to vehicle treatment. β-Actin was used as a protein loading control. Lower panels are representative immunoblots for the indicated proteins in the indicated Tg mice. Date are shown as mean ± s.e.m., *n* = 3 per group (one-way ANOVA in **e–j**)

We then evaluated the effect of overexpressing EP on cerebral Aβ pathology in mAPP mice. We first measured cerebral Aβ levels in mAPP and Tg *Sh3gl2*/mAPP mice by enzyme-linked immunosorbent assay (ELISA) and immunoblotting. Notably, Aβ levels, including Aβ40 and Aβ42, were significantly elevated in the entorhinal cortex (AD-affected regions at very early stages of AD and proceeding hippocampus) of Tg *Sh3gl2*/mAPP mice as compared with mAPP mice at the age of 5–5.5 months (Fig. 10a, b). These results suggest that overexpressing EP exacerbates cerebral Aβ accumulation. Given that overexpression of EP augmented ROS production and activation of p38 MAP kinase in mAPP mice, we examined whether EP-mediated oxidative stress and p38 MAP kinase signaling contributes to amyloid pathology. Intriguingly, administration of the ROS scavenger EUK-134 or the p38 MAP kinase inhibitor to Tg *Sh3gl2*/mAPP mice almost abolished elevated Aβ levels compared to vehicle treatment (Fig. 10c, d), suggesting that EP-induced oxidative stress and p38 MAP kinase signaling pathway may be responsible for cerebral Aβ accumulation. Immunoblots also confirmed elevation of Aβ levels in Tg *Sh3gl2*/mAPP mice compared to mAPP mice (Fig. 10e). We also found that the levels of β-site APP cleaving enzyme 1 (β-secretase 1, BACE1), critical to the generation of Aβ from APP, were significantly increased in Tg *Sh3gl2*/mAPP brain compared to mAPP brain (Fig. 10g). Increased levels of Aβ or BACE1 were suppressed by the antioxidant EUK-134 or p38 MAP kinase inhibitor SB203580 (Fig. 10f, h) treatment in Tg *Sh3gl2*/mAPP mice. Furthermore, increased EP reduced expression levels of insulin degrading enzyme (IDE), an enzyme for degrading Aβ to facilitate Aβ clearance (Fig. 10i). Similarly, administration of EUK-134 or SB203580 to Tg *Sh3gl2*/mAPP mice reversed IDE levels (Fig. 10j). These results indicate that increasing EP boosts Aβ production and accumulation possibly through APP processing or Aβ clearance by enhancing BACE1 activity and suppressing Aβ-degrading enzyme IDE. Together, these results suggest that EP-involved oxidative stress and the p38 MAP kinase signaling pathway are responsible for cerebral Aβ accumulation and production in Aβ-rich environment.

## Discussion

Synaptic dysfunction is an early marker in the progression of AD. However, the mechanisms of how this dysfunction occurs are only beginning to be identified. From studies into the proteomic consequences of Aβ binding to mitochondrial proteins, we have previously identified proteins that change their expression in dementias^38^, including synaptic proteins EF-hand domain-containing protein D2 (swiprosin-1), which is decreased^39^, and EP which is increased^28^. For the latter, we showed that EP protein levels were increased in the neurons of AD patients, and also in Tg animals with elevated Aβ levels^28^. Subsequently, we have reported that EP protein expression can be controlled by other factors, such as leptin^40^, while other groups have now associated EP with other dementias such as Parkinson’s disease^26^, spinocerebellar ataxia 2^41^, and Huntington’s disease^42^.

The consequence of this elevated EP level has been previously linked to the stress kinase pathways^28^. However, it is becoming increasingly clear that EP may have other direct effects on synaptic signaling as its expression level has been reported in several in vitro studies, to influence the probability of glutamate release^23^; brain-derived neurotrophic factor-activated tropomyosin receptor kinase B recycling^43^; and more recently influence spine formation^19^. Therefore, EP may have different effects in different locations, as it is found in both the pre-synapse and post-synapse^38^.

In this study, we sought to identify the in vivo consequences of a raised EP protein level in Aβ-rich environment to mimic EP levels in AD-affected brain. EP expression levels in mAPP brains were significantly elevated by 9–10 months of age (Supplementary Fig. 1b), suggesting that increased EP in mAPP mice could be a risk factor to promote/accelerate AD-like pathology such as mitochondrial and synaptic perturbation and amyloid pathology. To achieve this goal, Tg animals that overexpressed EP in neurons was produced in the presence of increased levels of Aβ. Levels of EP were elevated by 4–5-folds in mAPP brain (similar to the AD brain)^28^ compared to non-Tg brain; thus, these EP-overexpressing mice are an appropriate model for the study the effect of EP relevant to the AD. Notably, when EP overexpressed hippocampal slices expressing endogenous human Aβ or exposing exogenous Aβ, there were significant changes in LTP. This change in synaptic behavior was significant enough to change the cognitive function of these animals. To our knowledge, this is the first in vivo description that the elevated levels of EP in AD mice could have a significant effect on cognitive function.

Mitochondria are a major source of ROS generation. Inhibition of the electron transport chain by blocking complex activity in general would produce ROS. Aβ is capable of blocking the respiratory chain including complex I and IV^11,13,44–46^. In line with this, there is a significant decrease of mitochondrial complex IV activity in the AD-affected brains^44^, AD cybrid cells neurons containing AD-derived mitochondria or mild cognitive impairment-derived mitochondria, and Aβ-insulted neurons along with increased levels of mitochondria-derived ROS^36,45–47^. Deficiency in this key electron transport enzyme could lead to an increase in ROS production and reduction in energy stores. Indeed, suppression of ROS protected against EP/Aβ-induced mitochondrial and synaptic dysfunction as shown by restoring complex IV activity and ATP levels, increased LTP, reduced synaptic loss, and deficits in synaptic vesicle release both from in vitro cellular and in vivo EP/Aβ mouse models. Addition of the complex IV inhibitor KCN significantly reduced LTP and impaired synaptic vesicle release (Supplementary Figs. 8 and 9). Scavenging mitochondrial ROS by TEMPO restored the KCN-mediated synaptic deficits (Supplementary Fig. 9). Taken together, we believe the enhancement of ROS production in our animal model and the Aβ-treated ex vivo or in vitro models is contributed by disruption of the respiratory chain activity, such as complex IV. Given that mitochondria-derived ATP is important for maintaining normal synaptic function and vesicle cycling^48^, EP/Aβ-mediated ROS overproduction and the decline in synaptic mitochondrial ATP could contribute to synaptic dysfunction and aberrant synaptic vesicle cycling.

ROS production has been linked to the activation of the stress kinases^36,45^. We had previously shown that an increase in EP expression can activate the stress kinase. Here, we found a significant increase in p38 MAP kinase activity in the presence of elevated EP and Aβ-rich environment. Significantly, scavenging ROS production or blocking p38 MAP kinase activation not only reduced ROS levels but also restored mitochondrial function in the presence of Aβ, suggesting that EP-mediated p38 MAP kinase activation is responsible for Aβ-induced mitochondrial dysfunction and oxidative stress. Furthermore, inactivation of p38 MAP kinases alleviated the EP/Aβ-induced synaptic loss and deficits in vesicle recycling and hippocampal LTP, and improved learning and memory, indicating the impact of p38 MAP kinase activity on synaptic formation and function^49^.

Aβ can directly or indirectly mediate synaptic dysfunction through disruption of signal transduction including the PKA/CREB pathway^50^ or activation of p38 MAP kinase^51^. Although synaptic *N*-methyl-d-aspartate receptor is important for LTP, extra-synaptic NMDARs can trigger de novo long-term depression (LTD)^52^. Increasing the activation of NR2B-containing extra-synaptic NMDARs facilitates hippocampal LTD^53^. Aβ-inhibited LTP was prevented using selective NR2B inhibitors^54^. Thus, endophilin-mediated ROS production and p38 MAP kinase activation could be involved in NR2B-linked synaptic deficits. Immunoblotting of hippocampal lysates for NR2B exhibited a significant reduction in NR2B levels in Tg *Sh3gl2* hippocampal slices as compared with non-Tg hippocampal slices in the presence of Aβ (Supplementary Fig. 6a). Suppression of mitochondrial ROS or inactivation of p38 MAP kinase completely rescued the loss of the NR2B protein (Supplementary Fig. 6b), suggesting that an EP/ROS/p38 MAP kinase signal also contribute to NR2B-mediated synaptic damage insulted by Aβ.

Intriguingly, synaptic mitochondria are more sensitive to Aβ than soma-derived mitochondria^14^, and raised EP protein levels have been shown to be due to elevated levels of mitochondrial Aβ and its binding to mitochondrial protein amyloid binding alcohol dehydrogenase/17β hydroxysteroid dehydrogenase type 10 ^28^. Therefore, we hypothesize that an increase in mitochondrial Aβ leads to an increase in EP, which in turn leads to an increase in ROS production that stimulates p38 MAP kinase activity, subsequently disrupting the synaptic activity (both in changes in morphology and LTP), that then manifests itself in changes in cognition and behavior. Indeed, the addition of antioxidants and the specific p38 MAP kinase inhibitor not only suppresses ROS but also reverses Aβ-induced mitochondrial defects and activation of p38 MAP kinase signal transduction. Future studies will therefore focus on how an elevated EP protein level increases ROS production, presumably by affecting mitochondrial function, and as such this could be a previously unreported positive feedback mechanism to enhance the mitochondrial dysfunction that first caused the elevation of EP protein levels. Another intriguing possibility will be to explore the role of Parkin, which can bind to EP and can used as an ubiquination substrate, which is controlled by phosphorylation^26^. Disruption of both Parkin and Pink1-involved mitochondrial quality control could lead to the production of ROS^15,55^. In addition, EP has been reported to bind to LRRK2, another protein implicated in Parkinson’s disease^25^. LRRK2 activity has also been linked to mitochondrial activity; specifically, mutations in LRRK2 are linked to mitochondrial depolarization^55^.

Taken together, we have provided substantial evidence of the connection of EP with Aβ-induced alterations. First, we have demonstrated that overexpressing EP increased ROS levels and promoted mitochondrial dysfunction in in vitro hippocampal culture neurons and in vivo Tg *Sh3gl2*/mAPP mice, suggesting a link of EP to ROS and mitochondrial stress. ROS scavengers almost completely suppressed EP/Aβ-induced phosphorylation of p38 MAP kinase. Accordingly, the suppression of EP-induced activation of p38 MAP kinase increased complex IV activity and ATP levels, demonstrating a link between EP-induced ROS to p38 MAP kinase activation. Second, we provide evidence of the contribution of EP/ROS/p38 MAP kinase signaling to Aβ-mediated synaptic defects. Administration of an antioxidant or p38 MAP kinase inhibitor to Tg *Sh3gl2*/mAPP mice or hippocampal neurons alleviated LTP decline and synaptic loss, increased synaptic vesicle recycling, and improved learning and memory; these results indicate that blocking EP-involved ROS production and p38 MAP kinase activation restores synaptic and cognitive function in an model of AD-expressing EP/Aβ. Thus, EP/ROS/p38 MAP kinase signaling contributes to synaptic and cognitive perturbation in Aβ-rich environment. Finally, we observed exciting data on the promotion of cerebral Aβ accumulation and production in Tg *Sh3gl2*/mAPP mice. We have identified that EP also alters APP processing and Aβ clearance by the upregulation of BACE1 levels and the decrease in expression of the Aβ- degrading enzyme IDE. Notably, inhibition of EP-induced ROS or p38 MAP kinase activation blocked these increased levels of Aβ and BACE1, and restored IDE levels. Given that the detrimental effect of oxidative stress on the activity of α-secretase while elevated the expression and activation of β-secretase and γ-secretase, enzymes responsible for the generation of Aβ from APP^56–60^, our results indicate a contribution of EP/ROS/p38 MAP kinase signaling to amyloid pathology and abnormal Aβ/APP metabolism, possibly through APP processing and Aβ clearance.

Therefore, we report a new mechanism of how the synaptic protein, EP, which is elevated in AD patients, can lead to synaptic and cognitive dysfunction. We propose that increased levels of EP in AD and in an Aβ-rich brain disrupt the mitochondrial respiratory chain by inhibiting complex IV activity and ATP production, leading to excessive ROS production and accumulation. Consequently, excessive ROS activates p38 MAP kinase signaling, which is important for maintaining synaptic and cognitive function. Thus, EP-mediated signal transduction via ROS/p38 MAP kinase axis contributes significantly to mitochondrial dysfunction, synaptic injury, and cognitive decline. Furthermore, EP/ROS-mediated signal transduction could enhance amyloid pathology. Thus, inhibition of ROS production will prevent the activation of p38 MAP kinase signal pathway and rescue detrimental phenotypes in AD-type mice. Indeed, EP/Aβ-induced ROS production and mitochondrial dysfunction is prevented by ROS scavenger and the p38 MAP kinase inhibitor. As we have previously shown that EP protein levels can be controlled by other factors^40^, methods for attenuating EP expression could be sought to be a potential drug target, as is the use of p38 MAP kinase inhibitors or ROS scavengers, which are also being sought to be used in neurodegenerative diseases.

## Methods

### Mice

All studies on mice were performed in accordance to the National Institutes of Health guidelines for animal care with the approval of the Institutional Animal Care and Use Committee of the University of Kansas-Lawrence. To generate Tg mice overexpressing EP in neurons, we created a Tg expression cassette bearing *Sh3gl2* gene coding for full-length mouse EP (Genebank Accession Number NM_019535) driven by a Thy-1 promoter. A schematic depiction of Tg cassettes is shown in Supplementary Fig. 2a. The construct was verified by DNA sequencing. The founders of Tg *Sh3gl2 mice* were identified as bearing the transgene by PCR analysis of tail genomic DNA using genotyping primers (5′- ATGTCGGTGGCAGGGCTG-3′ (forward) and 5′-CTAATGGGGCAGAGCAACCAG-3′ (backward)). Tg *Sh3gl2* mice were backcrossed 10 times into C57BL6/J mice and then cross-bred with mAPP mice overexpressing an mAPP (J-20 line, obtained from Jackson Laboratory) to generate double Tg mice expressing neuronal Tg *Sh3gl2* and mAPP/human Aβ (Tg *Sh3gl2*/mAPP), single Tg (*Sh3gl2*, or mAPP), and non-Tg littermate offspring.

### Pharmacological treatment

Human Aβ1–42 were purchased from GenicBio, catalog number A-42-T-1, and oligermic Aβ was freshly prepared as previously described^11,61^. Brain slices from 3-month-old non-Tg and Tg *Sh3gl2* mice or primary cultured cortical neurons (day 14 in vitro (DIV14)) were treated with various drugs. The final concentration of the drugs were as follows: SB203580 (1 µM), EUK-134 (500 nM), and MitoTEMPO (1 µM). Mice were intraperitoneally injected with SB203580 (0.5 mg/kg) or EUK-134 (2 mg/kg) for 3 weeks.

### Hippocampal/cortical neuronal culture

We prepared hippocampal neurons from day 1 non-Tg as described previously^13^, culturing neurons in neurobasal medium supplemented with 1× B27, 600 μM l-glutamine, and penicillin–streptomycin. At DIV14, neurons from both Tg mice were treated with 50 nM Aβ in neurobasal medium supplemented with 0.5× B27 for an additional 24 h, with or without EUK-134 (500 nM, Cayman Chemical), SB203580 (1 µM, EMD Chemicals, Inc.), or MitoTEMPO (1 µM, Sigma) pretreatment for 1 h before the addition of Aβ. Vehicle was used as a control in neurobasal medium supplemented with 0.5× B27 for 24 h.

### Evaluation of the intracellular ROS

Evaluation of intracellular ROS levels was accessed by EPR spectroscopy. Brain tissues or cultured neurons was incubated with CMH (cyclic hydroxylamine 1-hydroxy-3-methoxycarbonyl-2, 2, 5, 5-tetramethyl-pyrrolidine, 100 μM) for 30 min, and then washed with cold phosphate-buffered saline (PBS) for three times. The brain tissues and neurons were collected and homogenized with 100 μl of PBS for EPR measurement. The EPR spectra were collected, stored, and analyzed with a Bruker EleXsys 540×-band EPR spectrometer (Billerica, MA, USA) using the Bruker software Xepr (Billerica, MA, USA)^62^.

### CcO activity assay

CcO (complex IV) activity was spectrophotometrically determined using CcO Assay Kit (Sigma) as our previous study^13^. In brief, indicated brain perfusion slices or brain tissues from hippocampal regions of indicated mice were homogenized in the lysis buffer, incubated on ice for 15 min, and centrifuged at 12,000 × *g* for 10 min. Suitable volume of supernatants and enzyme solutions were added into 475-μl assay buffer. The reaction was triggered by the addition of 25 μl ferrocytochrome c substrate solution (0.22 mM) into the cuvette. The changes in absorbance of cytochrome c at 550 nm wavelength was recorded immediately using a kinetic program with 5 s delay, 10 s interval, and total 6 readings on an Ultrospect 3100 Pro spectrophotometer.

### Measurement of ATP level

ATP levels were determined using an ATP Bioluminescence Assay Kit (Roche) following the manufacturer’s instruction. Briefly, indicated brain perfusion slices or brain tissues from hippocampal regions of indicated mice were homogenized in the lysis buffer provided, incubated on ice for 30 min, and centrifuged at 12,000 × *g* for 10 min. ATP levels were then measured in the subsequent supernatants using Luminescence plate reader (Molecular Devices). A 1.6 s delay time after substrate injection and 10 s integration time were used.

### Immunoblotting analysis

The preparation of cortical tissue extraction for immunoblotting was followed by the method described in our previous study^63^. Protein extracts were subjected to 10% Bis-Tris gel (Invitrogen, Grand Island, NY, USA), incubated with 5% non-fat dry milk in TBST buffer (20 mM Tris-HCl, 150 mM NaCl, 0.1% Tween-20) for 1 h at room temperature, and then followed by the primary antibodies with gently shaking overnight at 4 °C. The primary antibodies used were as follows: anti-EP (Cat# 36-3000, Invitrogen), anti-phos-p38 (Cat# 612288, BD), anti-total-p38 (Cat# 9212, Cell signaling), anti-human Aβ 1-17 clone 6E10 (Cat# 9320-02, Signet), anti-synaptophysin (Cat# MAB5258; Chemicon), anti-synaptojanin 1 antibody (AC1), (Cat# MA3-936; Thermo Fisher), anti-NMDAR2B (Cat# ab81271, Abcam), anti-BACE1 antibody (Cat# ab108394, Abcam), and β-actin (Cat# A5441; Sigma-Aldrich). ImageJ software (National Institutes of Health, Bethesda, MD, USA) was used for quantification of intensity of the immunoreactive bands in the scanned blots.

### Immunohistochemistry staining

Brain slices from the indicated Tg mice were subjected to double immunostaining with rabbit anti-EP (Cat# 36-3000, Invitrogen) and mouse anti-MAP2 (1:5000, sc-33796, Santa Cruz Biotechnology) at 4 °C overnight, followed by the conjugation of goat anti-rabbit Alexa Fluor488 and goat anti-mouse Alexa Fluor594. The staining images were taken under a Leica confocal microscope and analyzed by Universal Metamorp Image Program.

### Aβ measurement

Brain cortical tissues were incubated and homogenized in 5 M guanidine HCl and 50 mM Tris-HCl (pH 8.0) overnight and then subjected to Aβ concentration detection using human Aβ1–40 and Aβ1–42 ELISA Kits (Invitrogen) following the manufacturer’s instructions^64^.

### Immunocytochemistry studies

We prepared hippocampal neurons from postnatal day 1 pups as described previously^13^, followed by culturing them in neurobasal medium (Life Technologies) supplemented with 1× B27 (Life Technologies), 600 μM l-glutamine (Life Technologies), and penicillin–streptomycin (Life Technologies). The neurons at DIV14 were treated with 50 nM Aβ in neurobasal medium supplemented with 0.5× B27 for 24 h with or without various drugs (vehicle group) (EUK-134, 500 nM or MitoTEMPO, 1 µM or SB203580, 1 µM) pretreatment for 1 h. After 24 h incubation, neurons were fixed with 4% ice-cold paraformaldehyde for 5 min, and then incubated with 0.1% Triton and 5% goat serum in PBS for 1 h at room temperature (RT). The following primary antibodies were incubated with neurons overnight at 4 °C: rabbit anti-synaptophysin IgG (1:5000, Dako) and mouse anti-MAP2 IgG, (1:10,000, Chemicon). The secondary antibodies including Alexa Fluor^®^ 594-conjugated goat anti-rabbit IgG and Alexa Fluor^®^ 594-488 goat anti-mouse IgG (1:1000, Invitrogen) were incubated with neurons for 1 h at RT. Immunoreactive products were developed by Vectashield mounting medium (H-1000, Vector Laboratories). Images were taken at equal exposure for all different groups at ×63 oil lens under a confocal microscopy (Leica) using Universal Metamorp Image Program. Quantification of synaptic density of cultured neurons was described^13,15,47,63^. The experiments were performed by investigators blinded to the information about genotype and treatment until completion of image analysis.

### Behavioral test

Mice were subjected MWM test as described in our previous studies^13,15^. Briefly, in spatial acquisition session, mice were trained for five consecutive days with four trial each mouse per day. On the last day, a probe trial was performed to assess the spatial memory of mice. Traces of mice were recorded, and data were analyzed by HVS water 2020. Investigators were blinded by mouse genotypes during behavioral test.

### LTP recording

Transverse hippocampal slices (400 µm) were cut from the mouse brain and maintained in an interface chamber at 29 ℃ and perfused with artificial cerebrospinal fluid (ACSF) continuously bubbled with 95% O_2_ and 5% CO_2_. The ACSF composition was: 124 mM NaCl, 4.4 mM KCl, 1 mM Na_2_HPO_4_, 25 mM NaHCO_3_, 2 mM CaCl_2_, 2 mM MgCl_2_, and 10 mM glucose. CA3-CA1 ffEPSPs were recorded from the CA1 region of the hippocampus by placing the stimulating electrode at the level of the Schaeffer collateral (SC) fibers, whereas the recording electrode was placed in the CA1 *stratum radiatum*. Extracellular responses were acquired using Clampex software 14.2 (Molecular Device) and a microamplifier (IE-210, Warner Instruments). BST was assayed by plotting the slopes of fEPSP against the amplitude of fiber volley to generate input–output relations. A 30-min baseline recording was established using low-frequency stimulation (0.033 Hz; 0.1 ms impulse duration) and then adjusted intensity that induced fEPSPs with ~30% of the maximal fEPSP amplitude. The LTP was induced using *θ*-burst stimulation (4 pulses at 100 Hz, with the bursts repeated at 5 Hz, and each tetanus, including three 10-burst trains separated by 15 s). Hippocampal slices from 5-month-old to 6-month-old EP or EP/mAPP mice were pretreated with SB203580 (1 µM) or EUK-134 (500 nM) 5 min before Aβ perfusion (100 nM for 20 min). Values of fEPSP slope were expressed as mean ± s.e.m. percentage change relative to their mean baseline amplitude.

### Synaptic vesicles recycling (FM1–43)

We analyzed endocytosis–exocytosis as a measure of synaptic bouton function^65,66^. This strategy is based on the uptake and unloading of the styryl dye FM1–43 (Molecular Probes, Invitrogen) by hippocampal neurons that are plated on coverslips at a density of 1 × 10^5^ cells per coverslip. Neurons were incubated for 10 min in a low-K^+^ buffer: 130 mM NaCl, 5 mM KCl, 1.2 mM NaH_2_PO_4_, 1.8 mM CaCl_2_, 10 mM glucose, and 25 mM HEPES, pH 7.4, and were then labeled with 10 mM FM1–43 dye for 1 min in high-K^+^ buffer containing 79 mM NaCl and 56 mM KCl, followed by a 5 min wash by perfusion with a Ca^2+^-free and then the low-K^+^ buffer to remove the surface-bound dye. Baseline measurements were then acquired over 30 s by perfusing with a low-K^+^ medium and then stimulating the cells for 5 min in a high-K^+^ medium, leading to dye unloading.

Time-lapse recordings of images were acquired at a rate of one frame every 10 s on a Carl Zeiss (Axiovert 200) microscope with incubation system (PeCon) to maintain differentiated neuronal cells at 37 °C during image collection. Excitation was provided by a 479 nm monochromator, and emitted light was collected using a fluorescein isothiocyanate filter. Fluorescent signals were quantified using the MetaMorph software.

### Post hoc immunocytochemistry

To identify the field analyzed in the functional (FM1–43) experiments, the chambers subjected to post hoc immunohistochemistry were marked to ensure their position. After FM1–43 unloading, cultured neurons were fixed with 4% ice-cold paraformaldehyde for 30 min and then permeabilized with PBS containing 0.1% Triton and 5% goat serum for 1 h at room temperature, followed by incubation with primary antibody: mouse anti-MAP2 (1:5000, sc-33796, Santa Cruz Biotechnology), followed by the conjugation of a goat anti-mouse antibody.

### Statistical analysis

Student’s *t* tests were performed for analysis and comparisons between two groups. One-way analysis of variance (ANOVA) was used for repeated-measures analysis and comparisons in four groups, followed by Fisher’s protected least significant difference for post hoc comparisons. *P* < 0.05 was considered significant. StatView statistics computer software was used. All data were expressed as the mean ± s.e.m.

### Data availability

All data generated or analyzed during this study are included in this published article (and its Supplementary Information file).

## Electronic supplementary material

Supplementary Information
