# Supplementary material for: Overexpression of endophilin A1 exacerbates synaptic alterations in a mouse model of Alzheimer’s disease
Source: Nat Commun. 2018 Jul 30;9:2968. doi: 10.1038/s41467-018-04389-0 (PMC6065365; doi:10.1038/s41467-018-04389-0)
Supplement: Supplementary file 1 — Supplementary Information [file 41467_2018_4389_MOESM1_ESM.pdf]

Supplementary Materials

Overexpression of Endophilin A1 exacerbates synaptic alterations in a mouse model of Alzheimer's disease

Qing Yu [1, 2#], Yongfu Wang [1#], Fang Du [1], Shijun Yan [1], Gang Hu [1], Nicola Origlia[3], Grazia Rutigliano[3], Qinru Sun [1], Haiyang Yu [2], James Ainge [4], Shi Fang Yan [1], Frank Gunn-Moore [5$] and Shirley ShiDu Yan [1$, *]

1. Department of Pharmacology and Toxicology and Higuchi bioscience Center, University of Kansas, Lawrence, KS 66047, USA;

2. State Key Laboratory of Oral Diseases, National Clinical Research Center for Oral Diseases, West China Hospital of Stomatology, Sichuan University, Cheng Du, 610041, China;

3. CNR, Institute of Neuroscience, 56124, Pisa, Italy;

4. School of Psychology and Neuroscience, University of St Andrews, St Mary's Quad Street, KY16 9JP, St Andrews, UK;

5.School of Biology, Medical and Biological Sciences Building, University of St Andrews, North Haugh Street, KY16 9TF, St Andrews, UK.

# These authors contributed equally to this work

$ Co-senior author

* Corresponding authors

Shirley ShiDu Yan, M.D

Departments of Pharmacology and Toxicology and Higuchi Bioscience Center,

School of Pharmacy, University of Kansas,

2099 Constant Ave.,

Lawrence, KS 66047, USA

E-mail : shidu@ku.edu

Tel: 1-785-864-3637

Supplementary Figure legends

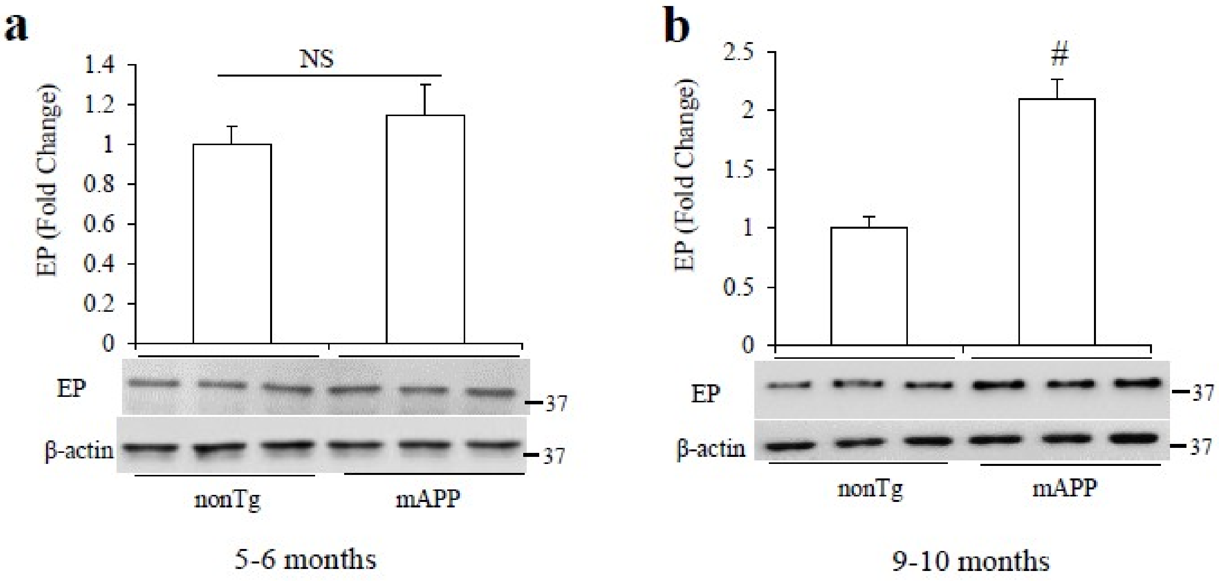

Supplementary Figure 1. **Expression levels of EP in mAPP mice.** Immunoblotting of brain homogenates show significant upregulation of EP levels the mAPP mice at 9-10 months but not at 5-6 months old. The representative immunoblotting for EP or β-actin were shown in the lower panel of **a-b**. Quantifications of the intensity of immunoreactivity bands for EP normalized by β-actin revealed an increased EP expression in 9-10 months old mAPP mice compared to nonTg mice in **b**, while there was no significant difference of EP expression between nonTg and mAPP mice at 5-6 months in **a**. Date are shown as mean ± s.e.m., n = 3 mice per group. NS, not significant (Student's t-test).

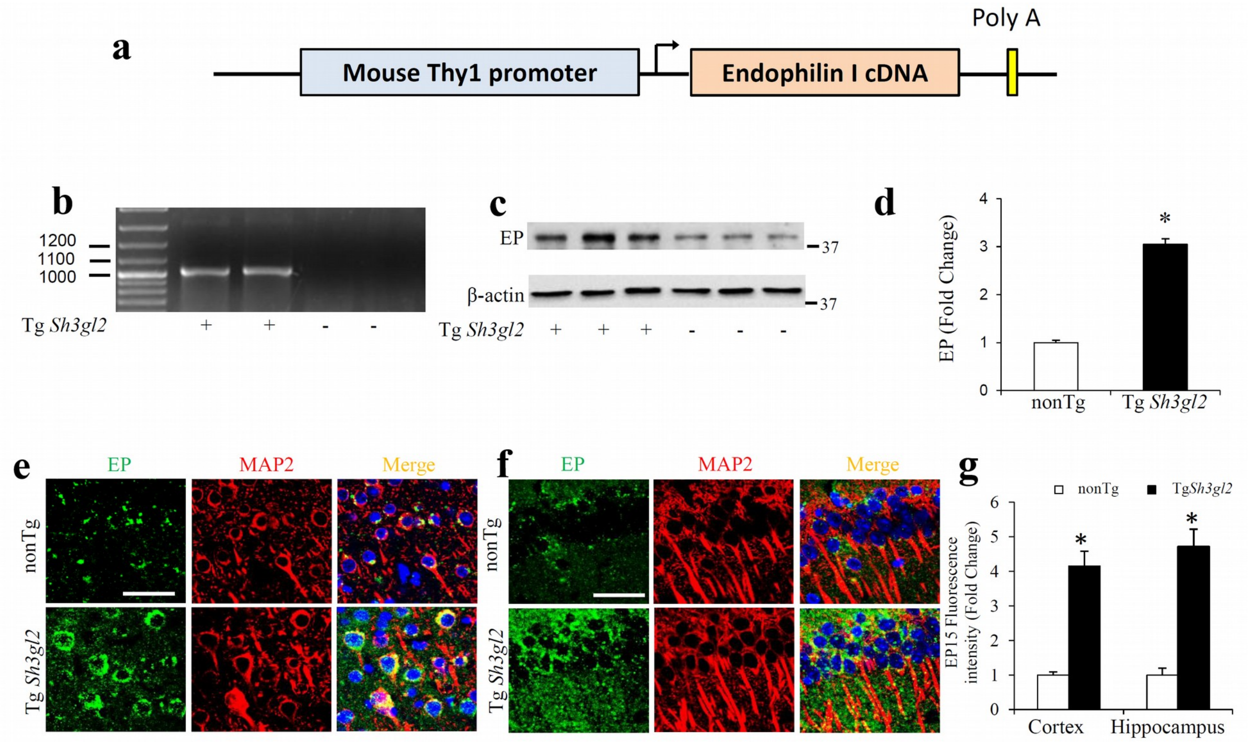

Supplementary Figure 2. Characterization of transgenic *Sh3gl2* mice. (a) Schematic depiction of transgenic cassettes for generation of transgenic (Tg) mice expressing neuronal endophilin A1 (EP) under the control of Thy-1 promoter. (**b)** PCR analysis of tail DNA was performed using the specific primers for *Sh3gl2* gene to identify Tg *Sh3gl2* (+) or non Tg mice (-) based on positive or negative PCR amplification. (**c-d**) Immunoblotting of brain homogenates from Tg *Sh3gl2*and nonTg mice were subjected to measurement of EP expression levels. The representative immunoblotting for EP or β-actin were shown in panel **c**. Quantification of intensity of immunoreactivity bands for EP normalized to β-actin revealed an increased EP expression in Tg *Sh3gl2* mice compared to nonTg littermate controls (**d**). (**e-f)** The representative immunostaining images of cortex (**e**) and hippocampus (**f**) from the indicated Tg mice for EP (green) and MAP2 (neuronal marker, red). (**g)** Quantification of the EP immunodensity (green) in cortex and hippocampus. Scale bars=25µm. Date are shown as mean ± s.e.m., n = 3 mice per group, * p < 0.01 compared to nonTg mice in **d** and **g** (Student's t-test).

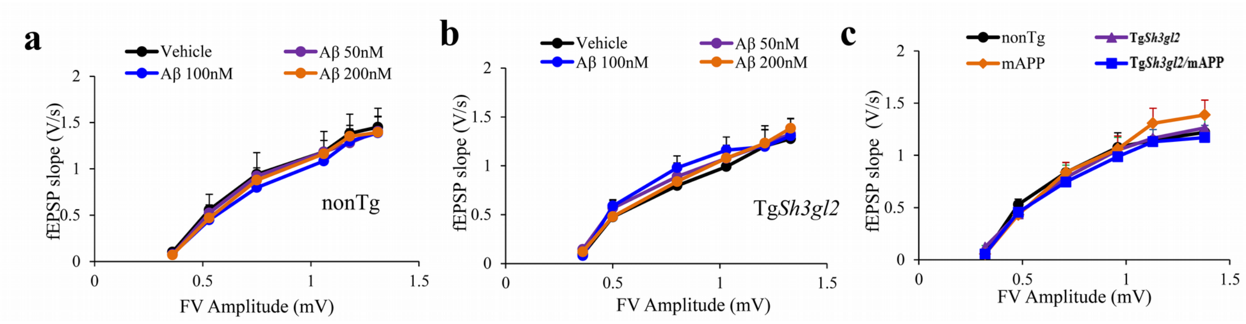

Supplementary Figure 3. The basic synaptic transmission in the indicated brain *in vivo* and brain slices *in vitro*. The basic synaptic transmission in nonTg hippocampus **(a)** or in Tg *Sh3gl2* hippocampus **(b)** was not altered in hippocampal slices perfused with 50 nM, 100 nM or 200 nM oligomer Aβ, as compared to vehicle treatment. **(c)** There were no significant differences in the basic synaptic transmission among the indicated groups of mice (nonTg, Tg *Sh3gl2*, mAPP and Tg *Sh3gl2*/mAPP). Error bars represent s.e.m., n=7-10 per group; No significant in **a-c** (One-way ANOVA).

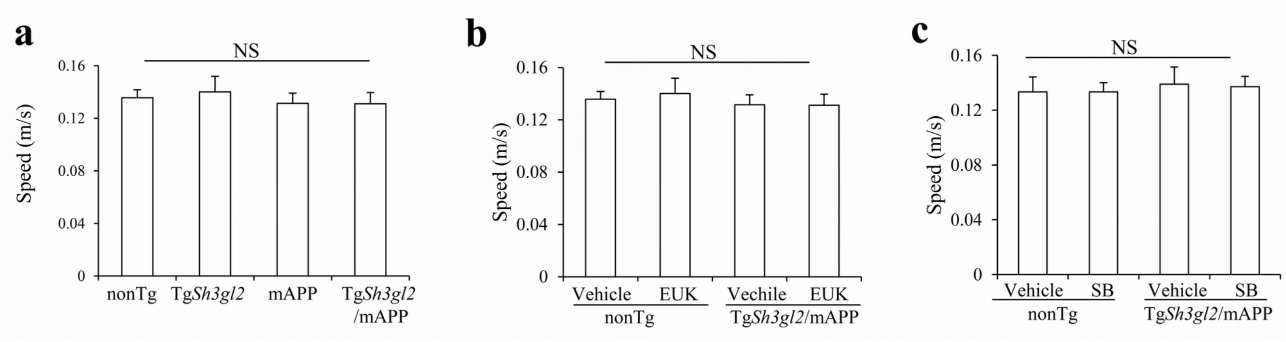

Supplementary Figure 4. Learning and memory by water maze swimming speeds in behavior test among Tg mice. The water maze test shows similar swimming speeds among nonTg, Tg *Sh3gl2*, mAPP and Tg *Sh3gl2*/mAPP groups (**a**), nonTg and Tg *Sh3gl2*/mAPP mice with or without EUK134 (**b**) or SB203580 treatment (**c**). Date are shown as mean ± s.e.m., n = 8-9 mice per group. NS, not significant (One-way ANOVA).

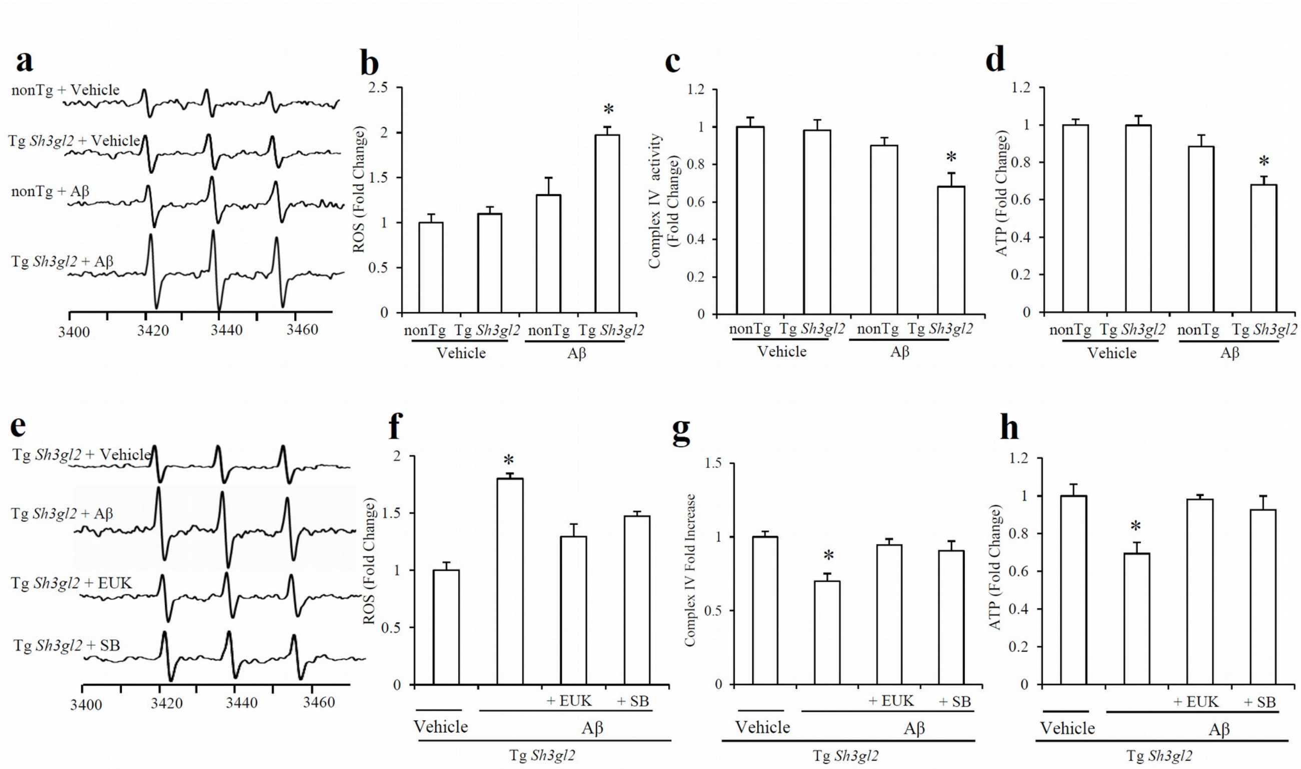

Supplementary Figure 5. Effect of antioxidant and p38 MAP kinase inhibitor on EP/Aβ-mediated ROS and mitochondrial dysfunction in cultured neurons. 14-day *in vitro* (DIV) cultured cortical neurons, either nonTg or Tg Sh3gl2, were treated with 50 nM Aβ for 24 hours, with/without 500 nM EUK-134 (EUK) or 1 μM SB203580 (SB) pretreatment for 1 hour before the addition of Aβ. **(a)** Representative spectra of EPR in Tg Sh3gl2 and nonTg neurons treated with Aβ (50 nM) for 24 hours. **(b)** Quantification of EPR spectra in the indicated groups of neurons. The peak height in the spectrum indicates the levels of ROS. Data are expressed as fold increase relative to nonTg vehicle treated neurons. **(c-d)** Mitochondrial complex IV activity and ATP levels were measured in nonTg or Tg Sh3gl2 neurons treated with vehicle or Aβ. Date are shown as mean ± s.e.m., n = 3-5 per group; * $p < 0.01$, compared to other groups (One-way ANOVA). (**e-f**) The treatment of either EUK-134 or SB203580 markedly reduced ROS level in Aβ-treated Tg Sh3gl2 neurons. The addition of EUK or SB203580 significantly increased mitochondrial complex IV activity **(g)** and ATP levels **(h)** in Aβ-insulted neurons. Only Tg

Sh3gl2 neurons with 50 nM Aβ treatment showed significant reductions in complex IV and ATP levels, and both were rescued by EUK or SB203580 pretreatment. Date are shown as mean ± s.e.m., n = 3-5 per group; * $p < 0.01$, compared to other groups (One-way ANOVA).

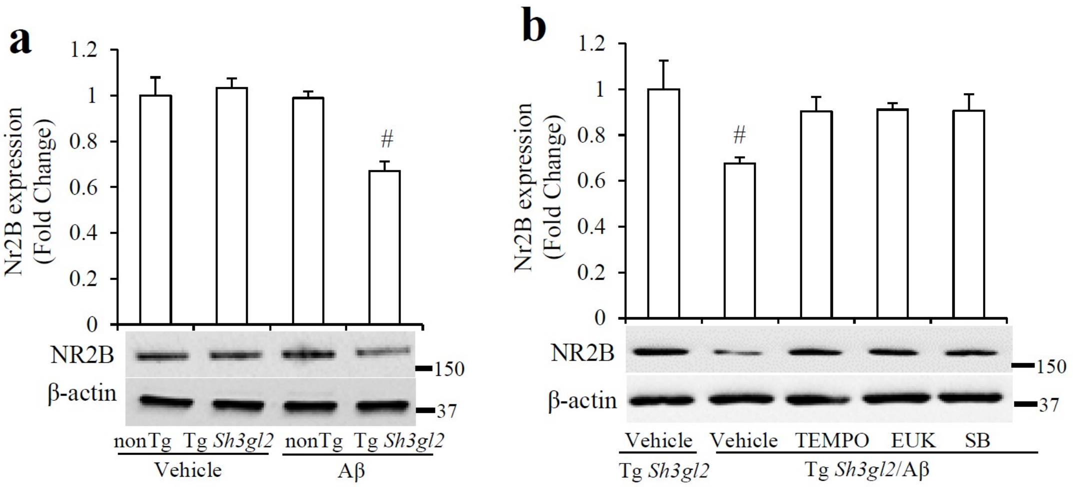

Supplementary Figure 6. Effect of EP on Aβ-induced NR2B expression in brain slice *in vitro*. (a) Brain slices from 3-month-old nonTg or Tg *Sh3gl2* mice were perfused with Aβ (50 nM) or vehicle for 2 hours, and then subjected to immunoblotting analysis for NR2B **(a)** in the indicated groups of brain slices. **(b)** The Tg *Sh3gl2* brain slices were perfused with Aβ (50 nM) for 2 hour with/without pretreatment of 500 nM EUK134 (EUK), 1 µM SB203580, or 1 µM MitoTEMPO for 5 min. Immunoblotting for NR2B in the indicated groups of brain slices. The upper panel displays the quantification of compared to other groups.Immunoreactive bands for the corresponding protein relative to β-actin. Data are expressed as fold increase relative to nonTg vehicle control group (**a**) or Tg *Sh3gl2* vehicle control group (**b**). Date are shown as mean ± s.e.m., n = 3 per group; $^{\#}p < 0.05$, compared to other groups (One-way ANOVA).

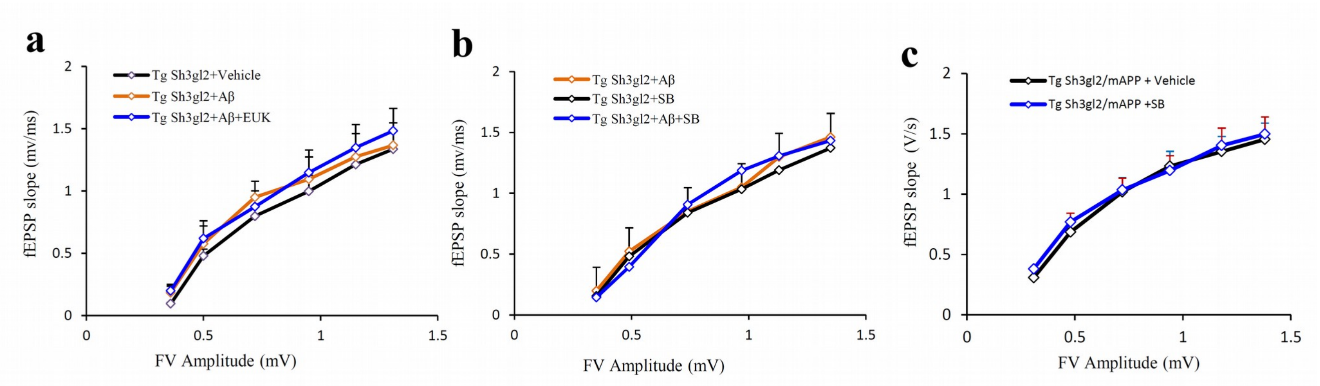

Supplementary Figure 7. The basic synaptic transmission in the indicated brain *in vivo* and brain slices *in vitro*. (a) The basic synaptic transmission in Tg *Sh3gl2* hippocampus was not altered in hippocampal slices perfused with 100 nM oligomer Aβ for 20 minutes with or without EUK pre-treatment, for 5 minutes, as compared to vehicle treatment. **(b)** The basic synaptic transmission in Tg *Sh3gl2* hippocampus was not altered in hippocampal slices perfused with 100 nM oligomer Aβ for 20 minutes with or without SB pre-treatment for 5 minutes, as compared to vehicle treatment. **(c)** There were no significant differences in the basic synaptic transmission among the indicated groups of Tg *Sh3gl2*/mAPP mice with or without SB injection. Error bars represent s.e.m., n=7-10 per group; No significant in **a-c** (One-way ANOVA).

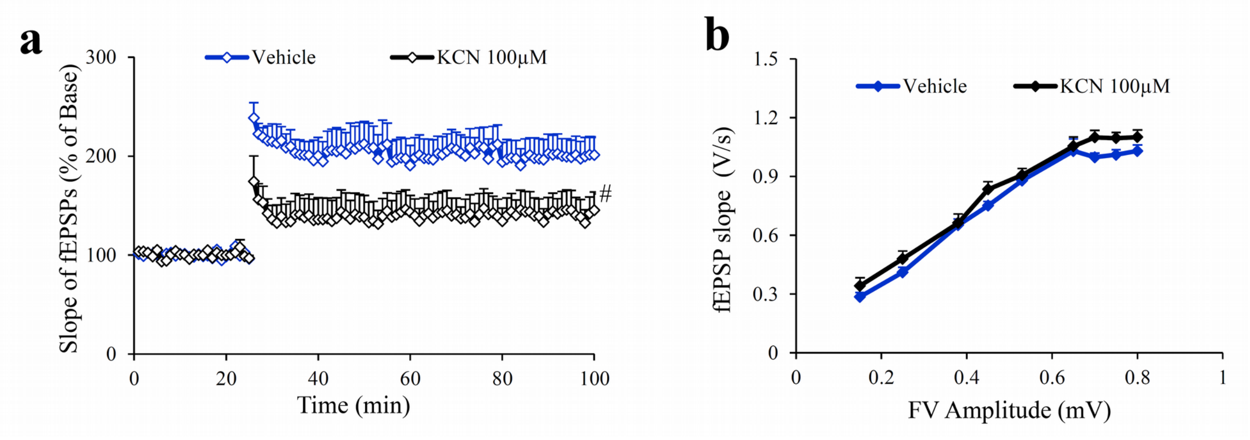

Supplementary Figure 8. Effect of complex IV inhibitor (KCN) on synaptic function in non-Tg mice. (**a-b**) Hippocampal slices from 3-4-month-old non-Tg mice, were treated with or without KCN (100 μM) and then hippocampal CA3-CA1 LTP was recorded **(a)**. The basal synaptic transmissions were unchanged in the indicated non-Tg hippocampal slices **(b)**. Error bars represent s.e.m., n=5 slices, 3 mice per group; $^{\#}p < 0.05$ compared to other groups (Student's t-test) in **a**. No significant (Student's t-test) in **b**.

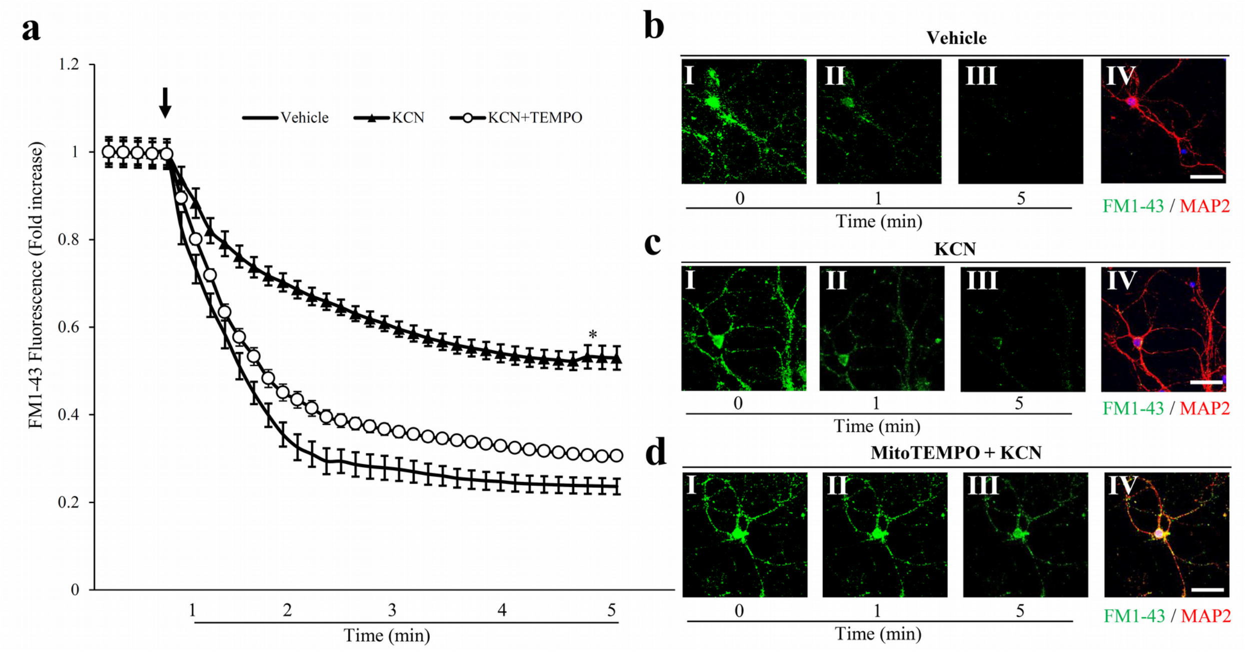

Supplementary Figure 9. Blocking oxidative stress in mitochondria rescued potassium cyanide (KCN)-induced synaptic vesicle recycling impairment. 14-day *in vitro* cultured cortical neurons were treated with 100 µM KCN for 24 hours, with or without 1 µM MitoTEMPO pretreatment for 1 hour, before the addition of KCN. To visualize synaptic vesicle recycling, the cells were loaded with the fluorescent styryl dye FM1-43 into synaptic vesicles and to unload this dye, after the stimulation with 50 mM K^+. (**a**) Kinetics of FM1-43 unloading of synaptic boutons during sustained stimulation with 50 mM KCl. (**b-d**) Fluorescence images before (**I**) and after (**II-III**) FM1-43 unloading with 50 mM KCl, and the representative immunofluorescence images of MAP2 (Red, **IV**) to ensure the position of FM1-43 fluorescence (Green, **IV**). Neurons were treated with 100 µM KCN for 24 hours alone (**c**), or with 1 µM MitoTEMPO pretreatment (**d**). Scale bars = 50 µm. Error bars represent s.e.m., n = 8 per group; $^*p < 0.01$ compared to other groups in **a** (One-way ANOVA).

Figure 4a

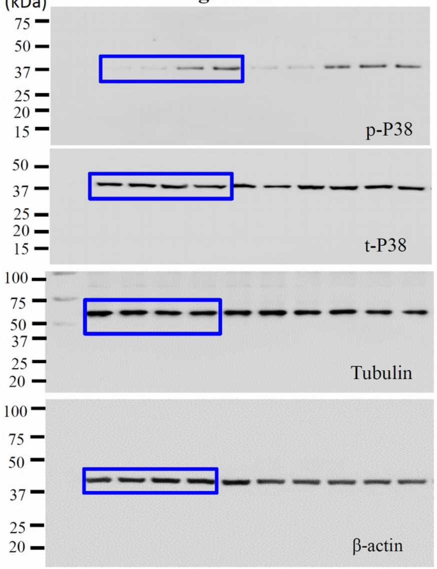

Figure 4b

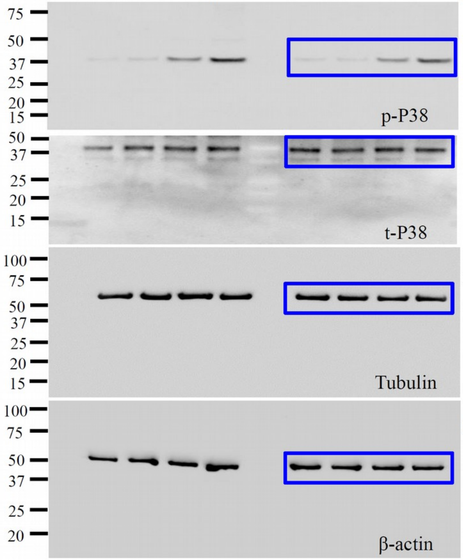

Figure 4c

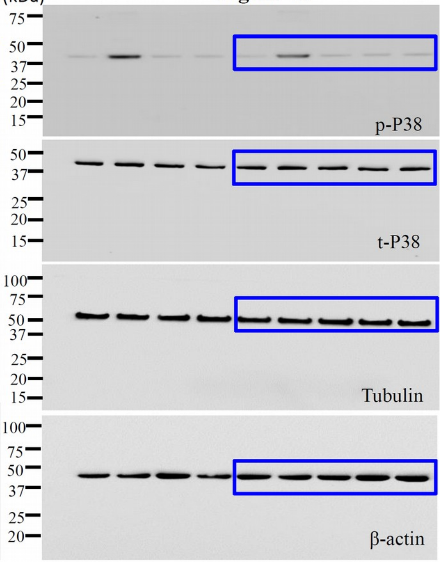

Figure 6a

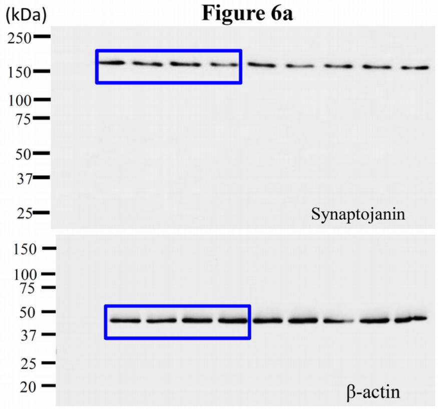

Figure 6b

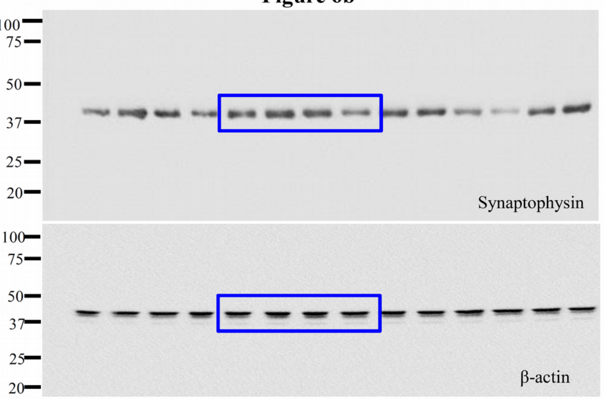

Figure 6c

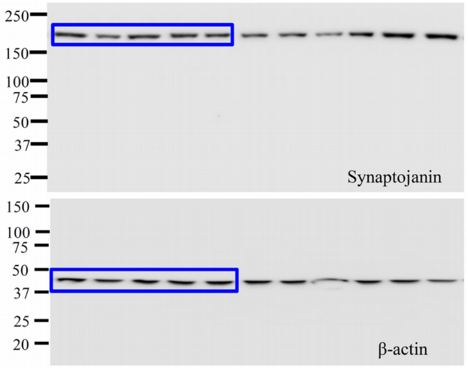

Figure 6d

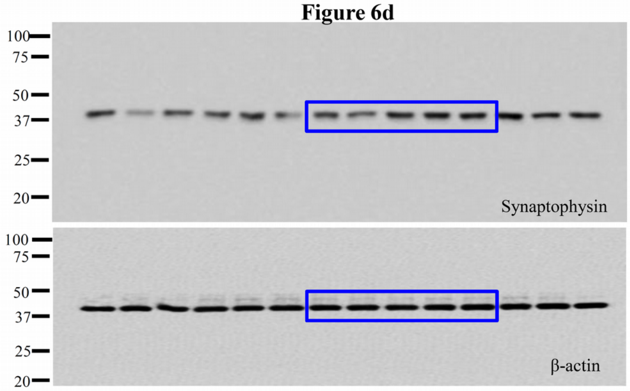

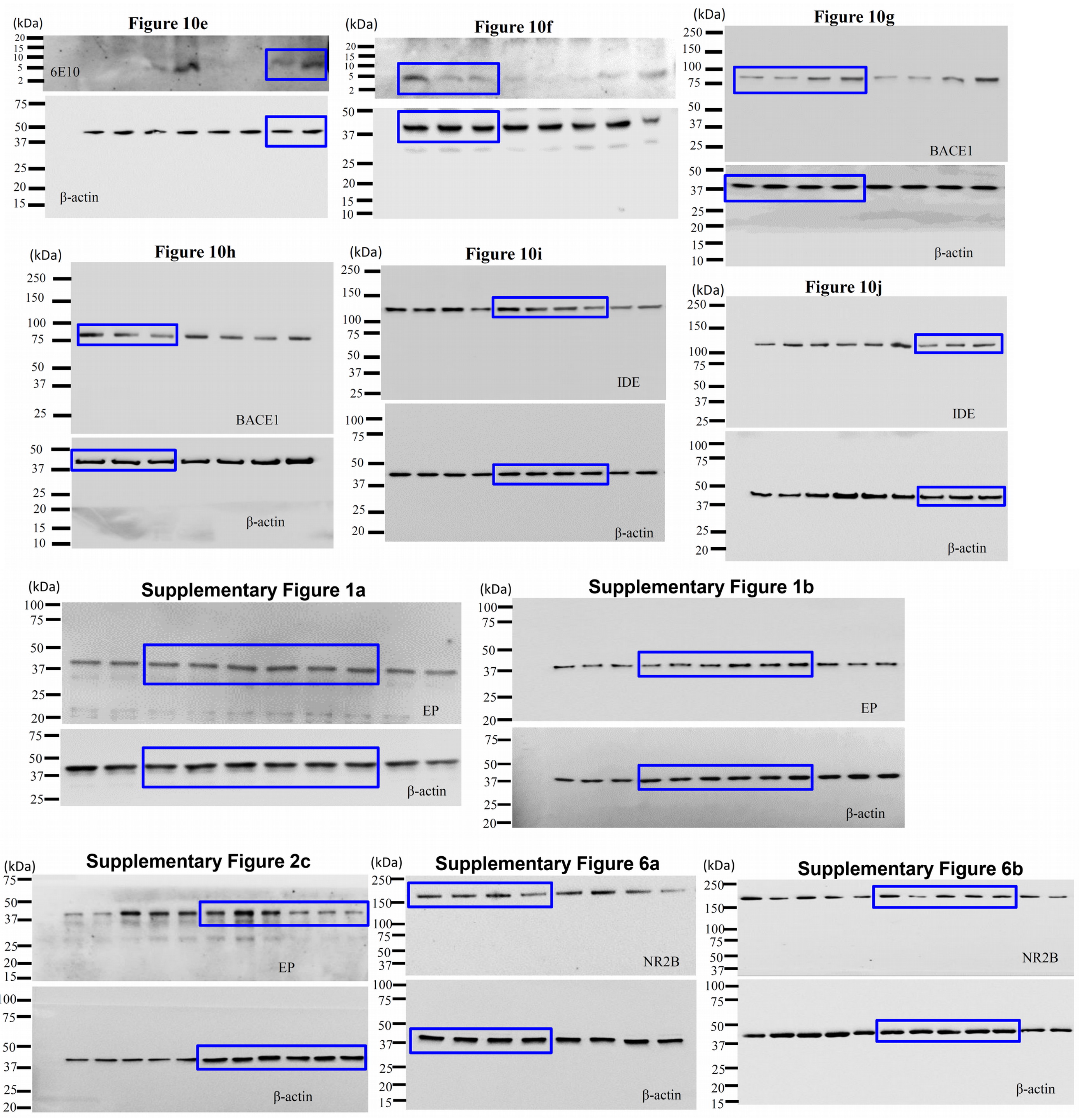

Supplementary Figure 10. Raw western blot data. Full scan of blots for main text figures **4a-c**, **6a-d**, **10e-j** and supplementary figures **1a-b**, **2c**, **6a-b**.
